# Pathological disruption of CELF2 shuttling causes neuronal hyperactivity, learning deficits, and seizures

**DOI:** 10.1172/JCI199698

**Published:** 2026-06-11

**Authors:** Michelle Hua, Mohamad-Reza Aghanoori, Melissa J. MacPherson, Yi Ren, Shehani V. Siripala, Yifan Yang, Yvonne Yan Yan Or, Malea Nguyen, Robert Duba-Kiss, Daniel Feng, Laura Williams, Christopher J. Gafuik, GengYi Wang, Chloe Quelin, Boris Keren, Sarah Schuhmann, Georgia Vasileiou, Alexia Bourgois, Antonio Vitobello, Christophe Philippe, Zornitza Stark, Richard J. Leventer, George McGillivray, Frederic Tran Mau-Them, Marine Tessarech, Clément Prouteau, Phillis Lakeman, Mahdi M. Motazacker, Donald R. Latner, Raymond C. Caylor, Yvette van Ierland, Eloise Prijoles, Angie Lichty, Evangelos Theodorou, David A. Sweetser, Edward Steel, Jan Cobben, Majed J. Dasouki, Daniel G. Calame, Bertrand Isidor, Benjamin Cogné, Mitchell Kesler, Brooke Rackel, Isabel Clark, Deborah M. Kurrasch, G. Campbell Teskey, James Ellis, Guiqiong He, Scott D. Ryan, Douglas J. Mahoney, A. Micheil Innes, Jonathan R. Epp, Guang Yang

**Affiliations:** 1 Department of Biochemistry and Molecular Biology and; 2 Department of Medical Genetics, Cumming School of Medicine, University of Calgary, Calgary, Alberta, Canada.; 3 Department of Medical Genetics, University of Alberta, Edmonton, Alberta, Canada.; 4 Department of Cell Biology and Anatomy;; 5 Department of Clinical Neuroscience, Cumming School of Medicine;; 6 Alberta Children’s Hospital Research Institute;; 7 Hotchkiss Brain Institute, Cumming School of Medicine; and; 8 Annie Charbonneau Cancer Institute, University of Calgary, Calgary, Alberta, Canada.; 9 Center for Neuroscience Research, School of Basic Medical Sciences, Chongqing Medical University, Chongqing, China.; 10 Service de Génétique Clinique, Centre de Référence «Anomalies du Développement et Syndromes Malformatifs» de l’Inter-région Ouest, CHU Rennes Hôpital Sud, Rennes, France.; 11 Department of Genetics, La Pitié-Salpêtrière Hospital, Assistance Publique Hospital of Paris, Paris, France.; 12 Sorbonne University, Paris, France.; 13 Institute of Human Genetics, Universitätsklinikum Erlangen, Friedrich-Alexander-Universität Erlangen-Nürnberg, Erlangen, Germany.; 14 Department of Human Genetics, Hannover Medical School, Hannover, Germany.; 15 Service de Génétique, Centre Hospitalier Universitaire de Caen, Caen, Basse-Normandie, France.; 16 Université Bourgogne Europe, CHU Dijon Bourgogne, Laboratoire de Génomique Médicale, Centre Neomics, FHU TRANSLAD, Centre de recherche Translationnelle en Médecine moléculaire – INSERM UMR1231, équipe GAD, Dijon, France.; 17 Laboratoire de Génétique, Hôpital de Mercy, CHR Metz-Thionville, Metz, France.; 18 Victorian Clinical Genetics Services, Murdoch Children’s Research Institute, Melbourne, Victoria, Australia.; 19 Department of Paediatrics, University of Melbourne, Melbourne, Victoria, Australia.; 20 Department of Medical Genetics, Angers University Hospital, Angers, France.; 21 Mitovasc Unit, UMR CNRS 6015 INSERM 1083, University of Angers, Angers, France.; 22 Service de Génétique Médicale, CHU d’Angers, Angers, France.; 23 Amsterdam UMC, Department of Human Genetics, University of Amsterdam, Amsterdam, Netherlands.; 24 Amsterdam Reproduction & Development Research Institute, Amsterdam, Netherlands.; 25 HudsonAlpha Institute for Biotechnology, Huntsville, Alabama, USA.; 26 Greenwood Genetic Center, Greenwood, South Carolina, USA.; 27 Department of Clinical Genetics, Erasmus University Medical Center, Rotterdam, Netherlands.; 28 Center for Genomic Medicine, Divisions of Pediatric Hematology/Oncology and Medical Genetics and Metabolism, Department of Pediatrics, Massachusetts General Hospital, Boston, Massachusetts, USA.; 29 Clinical Genetics, Great Ormond Street Hospital, London, United Kingdom.; 30 Section of Genomics and Genetics, Imperial College London, London, United Kingdom.; 31 AdventHealth Genomics & Personalized Health at Orlando, Department of Medical Genetics & Genomics, Orlando, Florida, USA.; 32 Section of Pediatric Neurology and Developmental Neurosciences, Department of Pediatrics, and; 33 Human Genome Sequencing Center, Baylor College of Medicine, Houston, Texas, USA.; 34 Jan and Dan Duncan Neurological Research Institute, Texas Children’s Hospital, Houston, Texas, USA.; 35 Nantes Université, CHU de Nantes, CNRS, INSERM, l’institut du thorax, Nantes, France.; 36 Nantes Université, CHU de Nantes, Service de Génétique médicale, Nantes, France.; 37 Department of Molecular Genetics, University of Toronto, Toronto, Ontario, Canada.; 38 Department of Microbiology, Immunology and Infectious Diseases, Snyder Institute for Chronic Diseases, and; 39 Owerko Center, University of Calgary, Calgary, Alberta, Canada.

**Keywords:** Clinical Research, Development, Genetics, Genetic diseases, Neurodevelopment, Seizures

## Abstract

De novo heterozygous variants in CUGBP Elav-like family member 2 (*CELF2*) have recently been associated with a rare neurodevelopmental disorder, yet the mechanisms linking specific variants to distinct clinical phenotypes remain poorly understood. Here, we reported a cohort of 18 individuals and provided evidence that variants causing CELF2 mislocalization, but not protein-null variants, were associated with seizures. Using proband-derived human cortical neurons and transgenic mouse models, we demonstrated that CELF2 underwent activity-dependent nucleocytoplasmic shuttling in excitatory neurons and that its cytoplasmic retention caused neuronal hyperactivity, elevated seizure susceptibility, and learning and memory deficits. We further found that cytoplasmic CELF2 regulated mRNAs critical for synaptic function and neuronal excitability and implicated in epileptic seizures and intellectual disability. Drug screening further identified AKT signaling as a key regulator of CELF2 nucleocytoplasmic shuttling and a candidate target for reversing neuronal hyperactivity. Together, our findings expand the clinical and genetic spectrum of CELF2-related neurodevelopmental disorders and establish a variant-specific mechanism that links CELF2 mislocalization to neuronal hyperactivity, seizures, and cognitive impairment.

## Abstract 2

Pathological mislocalization of CELF2 links neuronal hyperactivity to epilepsy and cognitive impairment

## Introduction

Mammalian CUGBP Elav-like family member 2 (*CELF2*) encodes a highly conserved multifunctional RNA-binding protein (RBP) (1). CELF2 contains 3 RNA recognition motifs and multiple regions resembling nuclear localization signals (NLSs) and nuclear export signals, allowing it to shuttle between the nucleus and cytoplasm and engage in multiple facets of RNA regulation (1–3). Nuclear CELF2 binds to introns in pre-mRNA to control alternative splicing, whereas cytoplasmic CELF2 modulates mRNA stability and translation in a target-specific manner (4–7). Therefore, alterations in CELF2’s subcellular distribution (7), expression (8), and RNA-binding activity (9) may have varying effects on gene expression, causing distinct functional outcomes. Others and we have previously reported individuals with de novo heterozygous missense variants that are exclusively clustered within and disrupt C-terminal NLSs, causing CELF2 mislocalization from the nucleus to cytoplasm without affecting its expression (7, 10). These individuals have a neurodevelopmental disorder (NDD) with features including seizures, global developmental delay, intellectual disability (ID), speech and language impairment, autism spectrum disorder (ASD), or brain malformations. Although the C-terminal NLSs partially overlap with RRM3, which is involved in RNA binding (3), mislocalized CELF2 mutant protein appears to retain its RNA-binding or splicing abilities (7). This suggests that the observed patient phenotypes are unlikely to result solely from a loss-of-function (LoF) mechanism. Instead, they may reflect a combination of cytoplasmic gain-of-function (GoF), a nuclear LoF, and/or other combinatorial effects due to CELF2 mislocalization, leading to phenotypic diversity. However, clinical and experimental evidence supporting the specific mechanisms of action of these variants in driving distinct phenotypic features is still lacking.

CELF2 is expressed throughout brain development, particularly in forebrain structures such as the cerebral cortex (7, 11, 12). Beyond its diverse effects on RNA, CELF2’s pleiotropic roles during brain development and the cell type–specific impacts of variants also likely contribute to the broad spectrum of neurodevelopmental symptoms. Our previous work demonstrates that in the embryonic mouse cortex, the dynamic nucleocytoplasmic shuttling of CELF2 is critical for maintaining neural precursor cell (NPC) homeostasis (7). By coordinating the translation of pro-differentiation mRNAs, CELF2 balances NPC proliferation and neurogenic differentiation, and its mislocalization disrupts this balance, causing abnormal development of the cortex (7). While these NPC defects may help explain certain features of cortical malformation, including macrocephaly and abnormal gyral patterns, other clinical phenotypes such as seizures, ID, and related learning deficits point to additional pathogenic mechanisms likely involving dysregulation of mature neurons (13, 14). In this regard, upon differentiation from NPCs in the mouse cortex, wild-type (WT) CELF2 relocates predominantly to the nucleus in newborn neurons, with its expression increasing substantially in excitatory neurons in the postnatal brain (7, 15). Further supporting the notion, a key feature of epilepsy is the hyperexcitability of excitatory neurons and dysregulated firing synchrony (14), often driven by pathological changes in genes encoding ion channels (e.g., *KCNA1/2*) (16), glutamate transporters (e.g., *SLC1A2*) (17), and synaptic proteins (e.g., *STX1B*) (18). Neuronal hyperexcitability can also disrupt synaptic dynamics and the coordinated activity of neuronal ensembles critical for encoding memory traces, leading to learning deficits associated with ID (19–21). Intriguingly, *Celf2*-knockout mice show normal spatial learning and memory but exhibit ASD-like behaviors, accompanied by reduced dendritic spine density and impaired synaptic maturation (8). These findings further suggest that CELF2 mislocalization-induced phenotypes may arise from mechanisms beyond LoF alone. However, the limited understanding of how CELF2 functions in neurons and the lack of information to stratify patient phenotypes make it difficult to ascertain the mechanisms that link CELF2 mislocalization to specific clinical presentations.

Here, we describe 18 individuals with heterozygous *CELF2* variants, including novel and recurring missense, nonsense, and frameshift variants that differentially led to nonsense-mediated mRNA decay (NMD), protein truncation, and CELF2 mislocalization. Phenotypic and functional assessments revealed that variants causing CELF2 mislocalization, but not protein-null variants, were associated with seizure presentation. Using patient-derived cortical neurons and genetically engineered mice, we found that CELF2 mislocalization, not LoF, caused neuronal hyperactivity, causing network dysregulation, elevated seizure susceptibility, failed separation of neuronal ensembles for learning tasks, and memory deficits in the fear conditioning test. In mice, mislocalized CELF2 proteins bound mRNAs encoding key regulators of synaptic functions and neuronal excitability implicated in epilepsy and ID. Moreover, CELF2 translocated between the nucleus and cytoplasm in response to neuronal activity, suggesting that its dynamic shuttling regulates activity-dependent plasticity of intrinsic excitability, with mislocalization variants disrupting this process and causing hyperactivity. Cell-based drug screening revealed that AKT signaling promoted cytoplasmic CELF2 translocation, and AKT inhibition reversed the hyperactivity defects. Our findings expand the clinical and genetic spectrum of CELF2-related NDD and highlight a critical role of dynamic CELF2 shuttling in regulating neuronal activity and brain functions. Moreover, our results indicate that variants disrupting CELF2 function in diverse ways can lead to distinct clinical manifestations, underscoring the need for tailored therapeutic strategies.

## Results

### Missense variants causing CELF2 mislocalization are associated with seizures.

**Figure 1:**
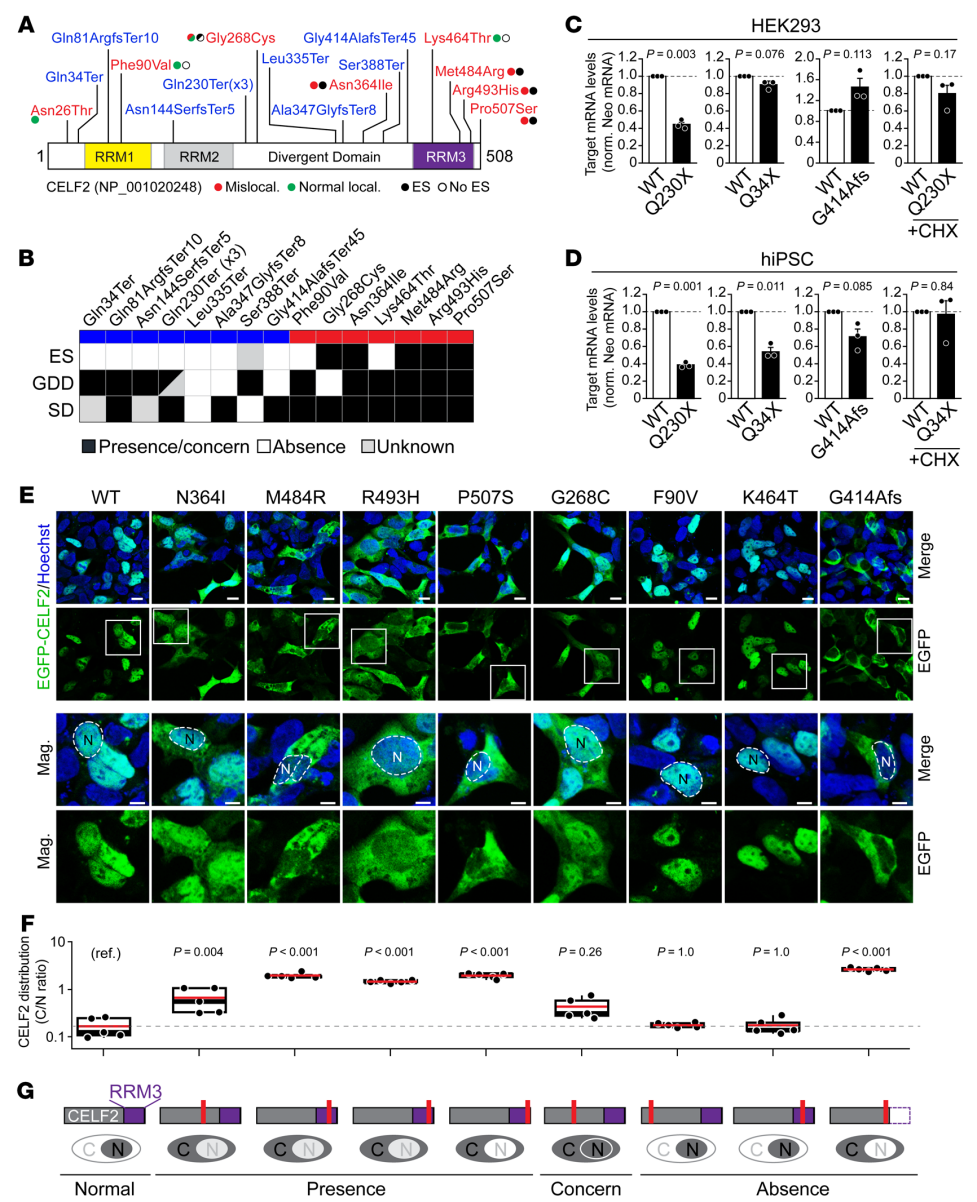
CELF2 missense variants causing CELF2 mislocalization are associated with epileptic seizures. (**A**) Schematic showing the positions of identified variants in the CELF2 protein, containing 3 RNA recognition motifs (RRM1–3). PTVs are shown in blue and missense variants in red. Colored dots indicate effect of missense variants on CELF2 subcellular localization; black circles denote seizure occurrence in corresponding individuals. (**B**) Heatmap of identified variants and associated clinical features, including ES (epileptic seizures), GDD (global developmental delay), and SD (speech delay). A feature’s presence is indicated by a black box; a gray box denotes unavailable information. (**C** and **D**) Bar graphs showing normalized mRNA levels of minigene reporters with the indicated variants, compared with WT, in HEK293 cells (**C**) or hiPSCs (**D**), treated with or without cycloheximide (CHX; 4 hours) as determined by qPCR. Data are presented as means ± SEM, normalized to WT. Each dot represents 1 experiment. One-sample 2-tailed *t* test. (**E**) Confocal images of HEK293 cells expressing WT EGFP-CELF2 (green) or the indicated variants. White box areas are shown at higher magnification below. Nuclei were counterstained with Hoechst 33258 (blue) and are outlined with dashed white lines. “N” denotes the nucleus. (**F**) Quantifications of cytoplasmic/nuclear ratio of CELF2 levels, from **E**. *n* = 5 (100 cells each). One-way ANOVA, Dunnett’s post hoc test, compared with WT. (**G**) Schematic of CELF2 protein variants tested in **E**, marked by vertical red bars, grouped by their subcellular localization pattern (nucleus “N” vs. cytoplasm “C”) and associated seizure status. Truncated protein lacking RRM3 is depicted without the domain. Scale bars, 5 μm.

Others and we previously identified heterozygous variants that perturb CELF2’s C-terminal NLSs (7, 10). Using the GeneMatcher platform (22), we subsequently identified an additional 18 individuals with heterozygous missense variants and protein-truncating variants (PTVs) across the *CELF2* gene, including 2 previously described recurring missense variants (p.Arg493His, p.Pro507Ser) (Figure 1A and Supplemental Figure 1, A and B; supplemental material available online with this article; https://doi.org/10.1172/JCI199698DS1). The genetic and clinical data are summarized in Supplemental Tables 1 and 2. Fifteen cases were de novo, and 2 were inherited from an affected father, with his variant inheritance status being unknown (Supplemental Figure 1B). The variants comprised 8 nonsense or frameshift variants and 7 missense variants, of which 4 were located around the C-terminal NLSs, 2 within the divergent domain between RRM2 and RRM3, and 2 in the N-terminal region, either within or adjacent to RRM1. One fetal case (p.Asn26Thr, 24-week gestational age) exhibited hydrocephalus, agenesis of the corpus callosum, and moderate ventricular dilatation. The remaining 17 individuals ranged from 1.5 to 36 years of age, with no significant sex differences (55.5% females). Affected individuals were described as having distinctive and overlapping NDD features, including global developmental delay, ID, speech delay, and epileptic seizures. ASD and other behavioral disorders were also diagnosed. Although NDD features were frequently observed within this cohort, we noticed that seizures were notably enriched among individuals with missense variants, particularly those clustering around the NLS region (Figure 1, A and B, and Supplemental Figure 1C).

All identified PTVs are predicted to trigger NMD, except p.Gly414AlafsTer45, which introduces a premature stop codon near the end of the second-to-last exon (Figure 1A). To test their effect on NMD, we employed minigene reporters containing the affected exons and flanking intron fragments, which can undergo effective splicing when expressed in HEK293 cells (Supplemental Figure 1, D and E). qPCR analysis revealed a significant reduction in mRNA levels from the p.Gln230Ter reporter compared with WT, which was abolished by translational inhibition with cycloheximide (CHX) or removal of the intronic sequences, confirming NMD activation (Figure 1C and Supplemental Figure 1, E and F). In contrast, p.Gln34Ter appeared to escape NMD (Figure 1C). In human induced pluripotent stem cells (hiPSCs), both variants reduced reporter mRNA levels (Figure 1D), suggesting cell type–specific NMD activation. As expected, p.Gly414AlafsTer45 did not affect reporter mRNA levels in either cell type (Figure 1, C and D), consistent with NMD escape and the production of truncated CELF2 proteins lacking the entire RRM3 and NLSs in the C-terminal region (Figure 1A and Supplemental Figure 1C).

To examine the effects of missense variants, we expressed each variant fused to the C-terminus of EGFP in HEK293 cells. While WT CELF2 was predominantly present in the nucleus, all missense variants, except p.Asn26Thr, p.Phe90Val, and p.Lys464Thr, showed varying degrees of cytoplasmic localization, which correlated with the presence and concern of seizures in individuals carrying these variants (Figure 1, A and E–G, and Supplemental Figure 1, G and H). Inclusion of previously identified variants in the analysis reinforced the connection between CELF2 mislocalization and seizure manifestations (Supplemental Figure 1, A and C). Notably, although the truncated p.Gly414AlafsTer45 protein was similarly mislocalized to the cytoplasm, the affected patient did not experience seizures (Figure 1E), suggesting an intact RRM3, alongside cytoplasmic mislocalization, may relate to the pathogenesis of epileptic seizures. Consistently, a previously reported seizure-associated variant p.Tyr508Ter preserves RRM3 and similarly causes CELF2 mislocalization (10).

### Cytoplasmic mislocalization of CELF2 causes neuronal hyperactivity and network dysregulation.

**Figure 2:**
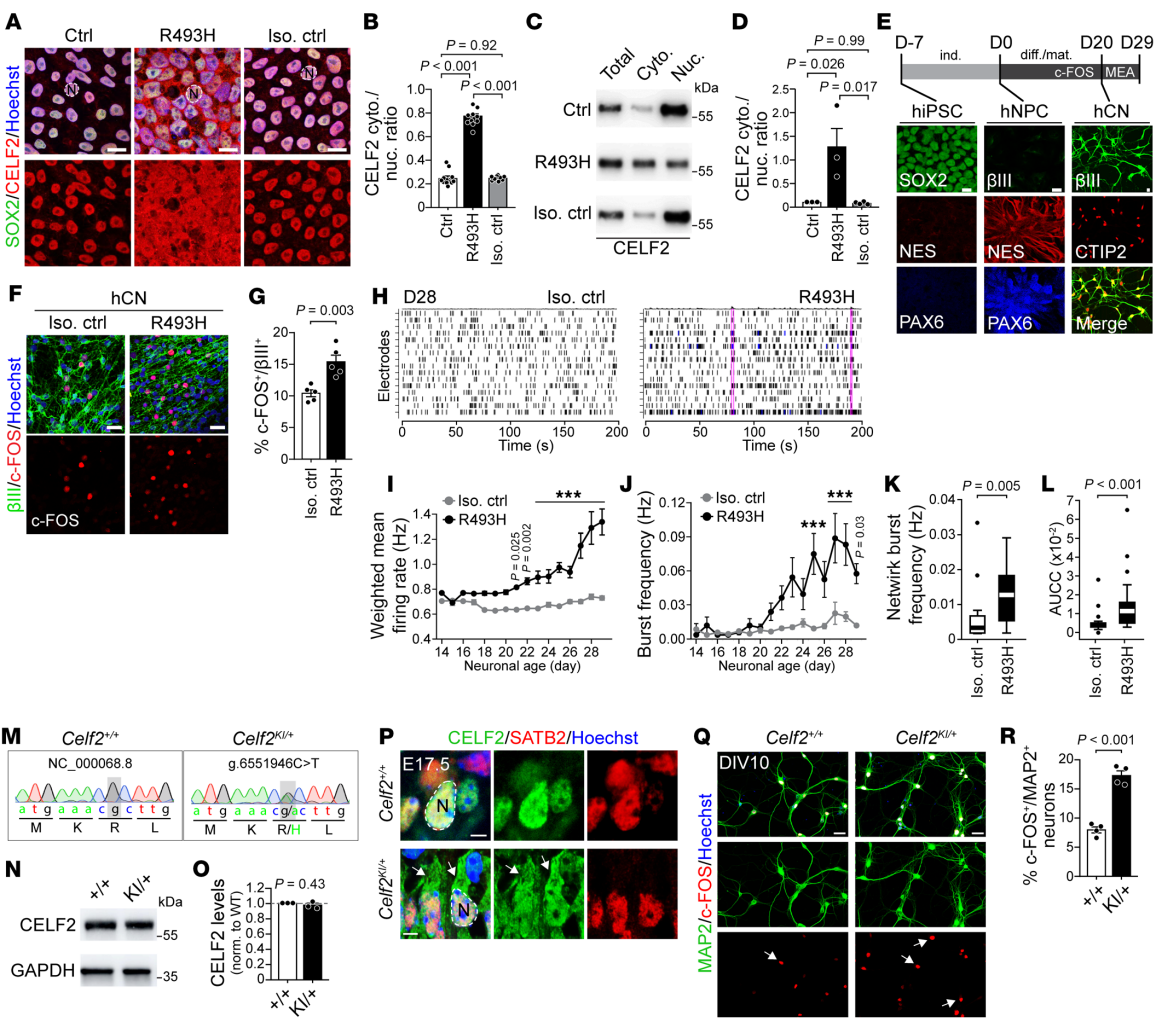
The p.Arg493His variant causes CELF2 mislocalization and neuronal hyperactivity. (**A**) Confocal images of control (Ctrl), p.R493H, and isogenic control (Iso. ctrl) hiPSCs, immunostained for CELF2 (red) and SOX2 (green). (**B**) Quantifications of CELF2 distribution, from **A**. *n* = 12 (600 cells each). (**C**) WB images and (**D**) quantifications of CELF2 levels in total lysates, cytoplasmic (Cyto.), and nuclear (Nuc.) fractions in hiPSCs. (**E**) Schematic and confocal images showing hiPSC induction into hNPCs for 7 days followed by differentiation into hCNs for 29 days, immunostained for indicated markers. diff., differentiation; ind., induction; mat., maturation. (**F**) Confocal images and (**G**) quantifications of βIII^+^ (green) and c-FOS^+^ (red) hCNs. *n* = 5 (2,000 cells each). (**H**–**L**) MEA of hCNs showing Raster plots of spike distribution on day 29 before and after electrical stimulations (**H**), weighted mean firing rate (**I**), and single-electrode burst frequency (**J**) over time and network burst frequency (**K**) and AUCC poststimulation (**L**). *n* = 17–24. ***P < 0.001. (**M**) Sanger sequencing of *Celf2^KI/+^* mice. (**N**) WB images and (**O**) quantifications of CELF2 levels in cortical lysates from E17.5 embryos. GAPDH serves as a loading control. (**P**) Confocal images of E17.5 cortex, immunostained for CELF2 (green) and SATB2 (red). Arrows highlight CELF2 signals in the cytoplasm. (**Q**) Confocal images and (**R**) quantification of MAP2^+^ (green) and c-FOS^+^ (red, arrows) neurons in mouse cortical cultures. Nuclei were counterstained with Hoechst 33258 (blue, **A**, **F**, **P**, and **Q**) and are outlined with dashed white lines in (**A** and **P**) with “N” denoting the nucleus. Means ± SEM. Each dot represents 1 experiment. One-way ANOVA, Tukey’s test (**B** and **D**); 2-way ANOVA, Šídák’s multiple comparisons test (**I** and **J**); Mann-Whitney *U* test (**K** and **L**); 1-sample 2-tailed *t* test (**O**); unpaired 2-tailed *t* test (**R**). Scale bars: 2.5 μm (**P**), 10 μm (**A** and **E**), 50 μm (**F** and **Q**).

Seizures are characterized by the hyperexcitability of neurons (14). To explore the mechanistic links between CELF2 mislocalization and seizures, we used hiPSCs derived from a patient carrying a recurring mislocalization missense variant (p.Arg493His) and isogenic hiPSCs in which the variant was corrected using CRISPR/Cas9-based gene editing (23). Immunostaining showed a substantially higher level of cytoplasmic CELF2 in p.Arg493His hiPSCs compared with both isogenic hiPSCs and control hiPSCs derived from a healthy individual (Figure 2, A and B, and Supplemental Figure 2A). This was corroborated by subcellular fractionation and Western blot (WB) analysis, which showed an 8-fold increase in the cytoplasmic-to-nuclear ratio of CELF2 in p.Arg493His hiPSCs (Figure 2, C and D, and Supplemental Figure 2B).

We then differentiated hiPSCs using the dual SMAD inhibition protocol and confirmed the expression of key marker genes at each stage, including NESTIN (NES) and PAX6 for early dorsal forebrain NPCs (hNPCs) and subsequent βIII-tubulin (βIII) and CTIP2 for cortical excitatory neurons (hCNs), with some cells beginning to express mature neuronal markers NCAM and MAP2 (Figure 2E and Supplemental Figure 2, C and D). Immunostaining for c-FOS, a marker of neuronal activation, revealed over 50% more c-FOS^+^βIII^+^ neurons in p.Arg493His cultures compared with the isogenic control (Figure 2, F and G, and Supplemental Figure 2, E–G), indicating hyperactivity of p.Arg493His hCNs.

During maturation, neurons integrate into networks with synchronized firing patterns, the dysregulation of which is linked to seizures (24). We examined network activity using multielectrode array (MEA) plates for differentiating hCNs over 29 days. While both WT and p.Arg493His hCNs showed increased spontaneous activity over time, the mutant cells had markedly higher weighted mean firing rate, burst number, and burst frequency, consistent with their hyperactive state (Figure 2, H–J, and Supplemental Figure 2H). To determine its impact on network connectivity and synchrony, we applied an electrical stimulation paradigm to hCN cultures on day 29 and measured evoked activity metrics. p.Arg493His hCNs produced substantially more network bursts at a higher frequency compared with WT, consistent with neuronal hyperexcitability (Figure 2K and Supplemental Figure 2, I and J). The evoked responses of mutant hCNs were also more synchronized, as evidenced by increased area under normalized cross-correlation (AUCC) (Figure 2L) and synchrony index (WT 0.008 vs. p.Arg493His 0.025). Despite greater activity and synchrony, mutant networks showed increased burst timing irregularity, as reflected by a concurrent elevation in inter-burst interval coefficient of variation (Supplemental Figure 2K), which suggests network instability. Collectively, these results suggest that p.Arg493His hCNs are hyperactive and exhibit epileptiform-like network activity.

To further investigate the effects of CELF2 mislocalization on neuronal activity, we generated knockin mice harboring 1 allele with the same p.Arg493His variant using the CRISPR/Cas9 approach (Figure 2M and Supplemental Figure 2L). WB analysis of cortical tissues from *Celf2^R493H/+^* mice (hereafter referred to as “KI”) showed no difference in CELF2 protein levels (Figure 2, N and O). However, we observed robust cytoplasmic CELF2 localization in SATB2-positive cortical excitatory neurons at E17.5, in contrast with the predominantly nuclear localization in their WT littermates (Figure 2P and Supplemental Figure 2M). This aligns with the mislocalization-inducing effect of the p.Arg493His variant. We then prepared primary neuronal cultures from the E17.5 cortex and performed immunostaining after 14 days in vitro (DIV), when neurons become mature enough to fire action potentials and show synchronized network activity (25, 26). In KI cultures, we detected approximately twice as many c-FOS–positive neurons as WT cultures (Figure 2, Q and R), phenocopying p.Arg493His hCNs.

### CELF2 mislocalization, not LoF, causes neuronal hyperactivity in mice.

**Figure 3:**
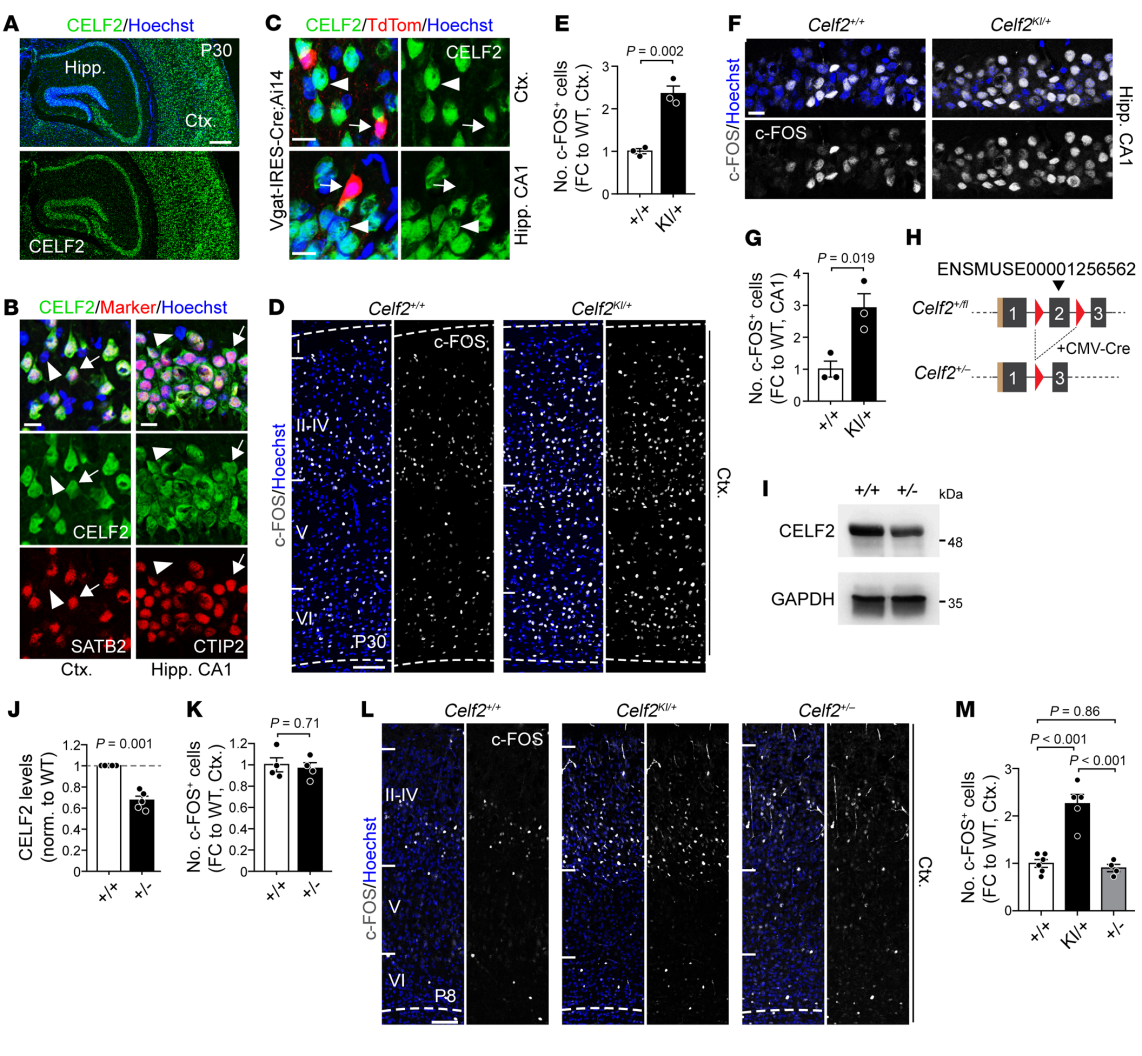
CELF2 mislocalization, not LoF, causes neuronal hyperactivity in mice. (**A**) Confocal images of cortical (Ctx.) and hippocampal (Hipp.) regions from P30 coronal brain sections, immunostained for CELF2 (green). (**B**) Magnified views from **A** showing CELF2 (green) in excitatory neurons, marked by SATB2 or CTIP2 (red). Arrows denote presence of markers; arrowheads, absence. (**C**) Confocal images of brain sections from a *Vgat-IRES-Cre* Ai14 reporter mouse, showing TdTomato-labeled inhibitory neurons (red, arrows) and the absence of CELF2 (green, TdTomato-negative cells [arrowheads]) in these cells. (**D**–**G**) Confocal images (**D** and **F**) of P30 cortical sections immunostained for c-FOS (white), with quantifications (**E** and **G**) of c-FOS^+^ cells in the cortical and hippocampal CA1 regions of *Celf2^KI/+^* mice, normalized to WT *Celf2^+/+^* mice. (**H**) Schematic showing the deletion of exon 2 in the *Celf2* gene, flanked by *loxP* sites (red triangles), by crossing a *CMV-Cre* deleter with a conditional knockout line (*Celf2^+/fl^*). (**I**) Western blots of cortical lysates from E17.5 WT and heterozygous knockout (*Celf2^+/–^*) mice, probed for CELF2 and GAPDH as a loading control. (**J**) Quantifications of CELF2 protein levels from **I**. (**K**) Quantifications of c-FOS^+^ cells in the P45 cortex of *Celf2^+/–^* mice, normalized to WT mice. (**L** and **M**) Confocal images (**L**) of P8 cortical sections immunostained for c-FOS (white), with quantifications (**M**) of c-FOS^+^ cells. Nuclei were counterstained with Hoechst 33258 (blue in **A**–**D**, **F**, and **L**). Means ± SEM. Each dot represents 1 animal or experiment. Unpaired 2-tailed *t* test (**E**, **G**, and **K**), 1-sample 2-tailed *t* test (**J**), and 1-way ANOVA, Tukey’s test (**M**). Scale bars: 10 μm (**B**, **C**, and **F**), 100 μm (**D** and **L**), 500 μm (**A**).

To ask whether CELF2 mislocalization affects excitatory neurons in their native environment, we examined KI mouse brains. Consistent with previous reports (7, 15), CELF2 was highly expressed in SATB2- and CTIP2-positive excitatory neurons in the postnatal cortex and hippocampus but low in inhibitory neurons labeled by GABA or TdTomato in a *Vgat-Cre* reporter line (Figure 3, A–C, and Supplemental Figure 3A), which aligns with the cell-autonomous effect observed in cultured neurons. Immunostaining at P30 revealed a 2~3-fold increase in c-FOS–positive cells in the KI cortex and hippocampus compared with WT littermates (Figure 3, D–G). This increase was not due to altered cell numbers, as cortical thickness and cell density were comparable between WT and KI mice (Supplemental Figure 3, B–D). At P45, when inhibitory neuronal circuits reach greater maturity (27–29), we likewise observed a robust increase in c-FOS–positive cells in both male and female KI mice (Supplemental Figure 3, E–I). Within cortical layers II–VI, all c-FOS^+^ cells coexpressed SATB2, consistent with an excitatory neuron identity (Supplemental Figure 3, J–L). Together, these findings suggest that CELF2 mislocalization alters the intrinsic firing properties of excitatory neurons.

Neuronal hyperactivity may be driven by nuclear LoF, cytoplasmic GoF, or a combination of both, resulting from CELF2 mislocalization. To explore these possibilities, we generated *Celf2* heterozygous knockout (*Celf2^+/–^*) mice, which exhibited an approximately 40% reduction in CELF2 protein levels (Figure 3, H–J). Intriguingly, the number of c-FOS^+^ neurons in the *Celf2^+/–^* cortex remained unchanged at P45, compared with WT (Figure 3K and Supplemental Figure 3M). Cortical excitatory neurons undergo rapid growth during early postnatal weeks, with c-FOS first detectable around P8 and steadily increasing over time (Supplemental Figure 3, N and O). Notably, elevated c-FOS^+^ neurons were already detectable in the KI cortex at P8, while *Celf2^+/–^* and WT mice showed no difference (Figure 3, L and M), suggesting that the observed neuronal hyperactivity likely reflects an effect of cytoplasmic GoF rather than reduced nuclear CELF2.

### CELF2 shows dynamic nucleocytoplasmic distribution during excitatory neuron maturation.

**Figure 4:**
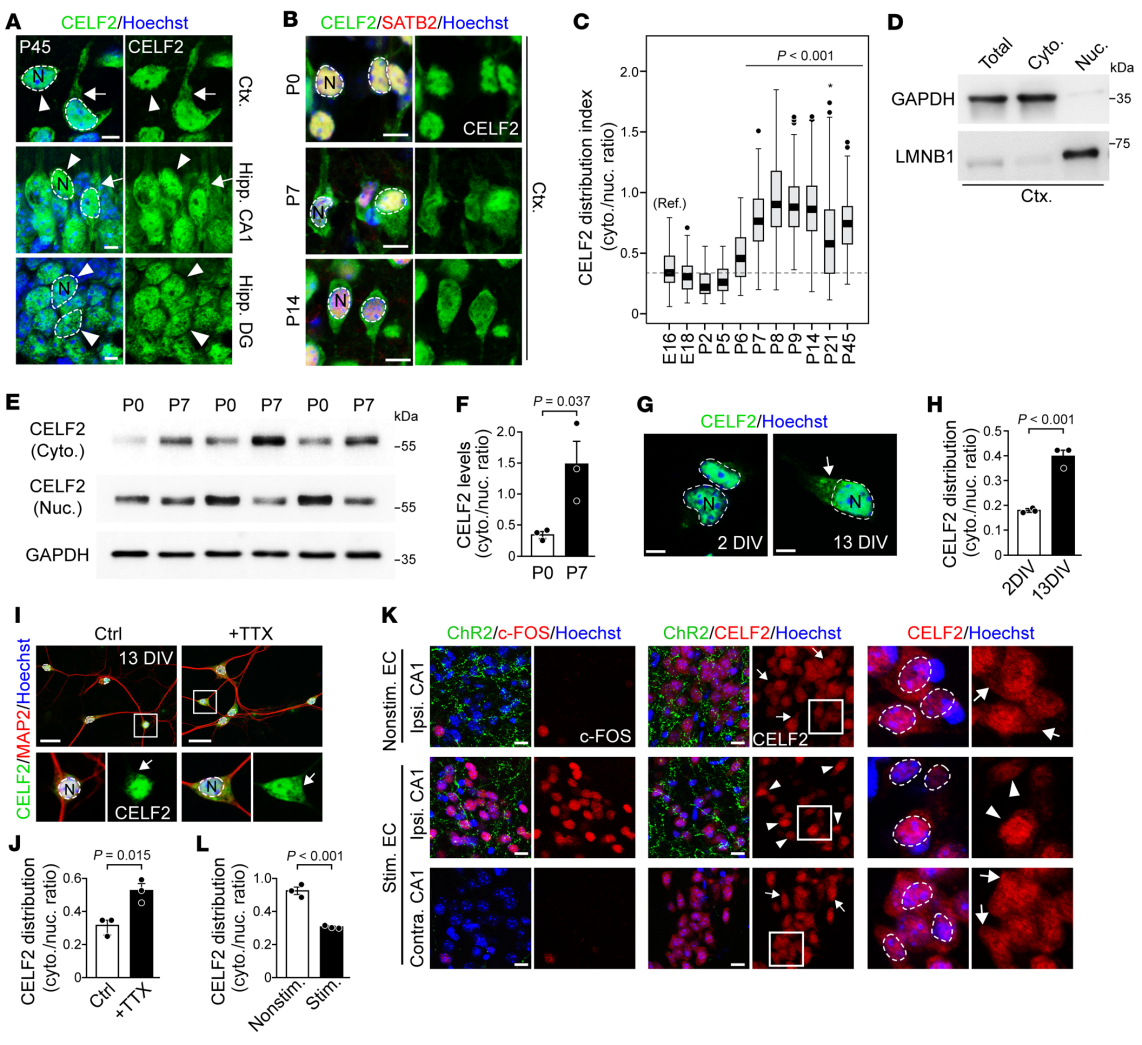
CELF2 shows dynamic subcellular localization in response to neuronal activity in mice. (**A**) Confocal images of cortical and hippocampal regions from P45 mouse brain sections, immunostained for CELF2 (green). (**B**) Confocal images showing CELF2 expression (green) in SATB2^+^ (red) cortical neurons from P0, P7, and P14. (**C**) Quantifications of CELF2 nucleocytoplasmic distribution in cortical neurons at the indicated developmental stages. *P* < 0.001 except for P6 (*P* = 0.03) and P21 (*P = 0.01). Mann-Whitney *U* test with Bonferroni-Holm correction, E16 as reference. *n* ≥ 30 cells each. Box plots show the interquartile range (IQR), median (line), and 1.5× IQR (whiskers). (**D**) WB images of total lysates, cytoplasmic (Cyto.), and nuclear (Nuc.) fractions from P7 cortical tissues, probed for GAPDH (cytoplasmic marker) and LMNB1 (nuclear marker). (**E**) WB images and (**F**) quantifications of CELF2 levels in cytoplasmic and nuclear fractions from P0 and P7 cortical tissues. (**G**) Confocal images and (**H**) quantifications of CELF2 (green) distribution in cortical neurons cultured for 2 or 13 DIV. *n* = 3 (≥30 cells each). (**I**) Confocal images and (**J**) quantification of CELF2 (green) distribution in MAP2^+^ (red) 13 DIV neurons treated with or without TTX for 48 hours. Magnified views of the white boxed area are shown below. *n* = 3 (≥35 cells each). (**K**) Confocal images and (**L**) quantification of CELF2 distribution in hippocampal CA1 regions ipsilateral or contralateral to AAV injection sites, with or without blue light stimulation. Sections were immunostained for EGFP-ChR2 (green) and c-FOS or CELF2 (both red). Magnified boxed areas are shown to the right. *n* = 3 (≥25 cells each). Means ± SEM. Each dot represents 1 sample. Hoechst-stained nuclei (“N,” blue, **A**, **B**, **G**, **I**, and **K**) are outlined with dashed white lines. Arrows and arrowheads (**A**, **G**, **I**, and **K**) highlight predominant cytoplasmic and nuclear CELF2, respectively. Unpaired 2-tailed *t* test (**F**, **H**, **J**, and **L**). Scale bars: 5 μm (**A** and **G**), 10 μm (**B** and **K**), 25 μm (**I**).

To investigate the role of cytoplasmic CELF2 in neuronal activity, we examined its subcellular distribution in mouse excitatory neurons. In the embryonic cortex, CELF2 was predominantly nuclear (Figure 2P). Surprisingly, in the P45 cortex and hippocampus, its localization varied, with some neurons retaining nuclear CELF2, while others showed variable cytoplasmic localization (Figure 4A). A similar pattern was observed in the human brain, where CELF2 also exhibited differential cytoplasmic localization in adult cortical and hippocampal neurons, distinct from the embryonic stage (Supplemental Figure 4, A–C). These findings indicate a developmental shift in CELF2 localization. Indeed, analysis of mouse cortical neurons across midembryonic to postnatal stages revealed an apparent transition around P5, with CELF2 shifting from predominantly nuclear to cytoplasmic and peaking at P8, followed by gradual divergence into subpopulations with varying levels of nucleocytoplasmic CELF2 localization (Figure 4, B and C). The transition in CELF2 localization during the perinatal stage was corroborated by fractionation analysis, which confirmed a marked increase in cytoplasmic CELF2 levels (Figure 4, D–F).

### CELF2 changes subcellular localization in response to neuronal activity in mice.

This initial nuclear-to-cytoplasmic shift of CELF2 localization in mouse cortical neurons preceded c-FOS onset, and its peak and subsequent divergence coincided with increasing c-FOS^+^ neuron numbers (Figure 4C and Supplemental Figure 3, N and O). Moreover, mature neurons in 14 DIV cultures showed higher cytoplasmic CELF2 levels than immature neurons at 2 DIV (Figure 4, G and H). These findings raise the possibility that CELF2 may change its localization in response to neuronal activity to modulate intrinsic excitability. To test this, we treated 14 DIV primary neuronal cultures with tetrodotoxin (TTX), a sodium channel blocker, for 48 hours, and found a significant increase in CELF2 cytoplasmic localization (Figure 4, I and J). Next, to test whether neuronal activation induces CELF2 nuclear translocation, we used an optogenetics approach to stimulate entorhinal cortex (EC) afferents, which project to hippocampal cornu ammonis 1 (CA1) pyramidal neurons. To this end, we injected adult mice with adeno-associated virus–ChR2-EGFP (AAV-ChR2-EGFP) into the lateral EC, followed 2 weeks later by 5-minute pulsed blue light stimulation. Immunostaining 90 minutes after stimulation showed robust c-FOS induction within the ipsilateral CA1 regions, alongside ChR2-EGFP expression. Importantly, these hyperactivated neurons showed greater nuclear CELF2 localization compared with nonstimulated neurons or contralateral CA1 neurons lacking ChR2-EGFP fibers (Figure 4, K and L). Our results indicate that cytoplasmic CELF2 promotes neuronal excitability, and its nuclear translocation in activated neurons may enable their adjustment of excitability. Disrupting this dynamic shuttling of CELF2 may contribute to neuronal hyperactivity.

### CELF2 p.Arg493His binds mRNAs implicated in epileptic seizures and regulators of neuronal excitability.

**Figure 5:**
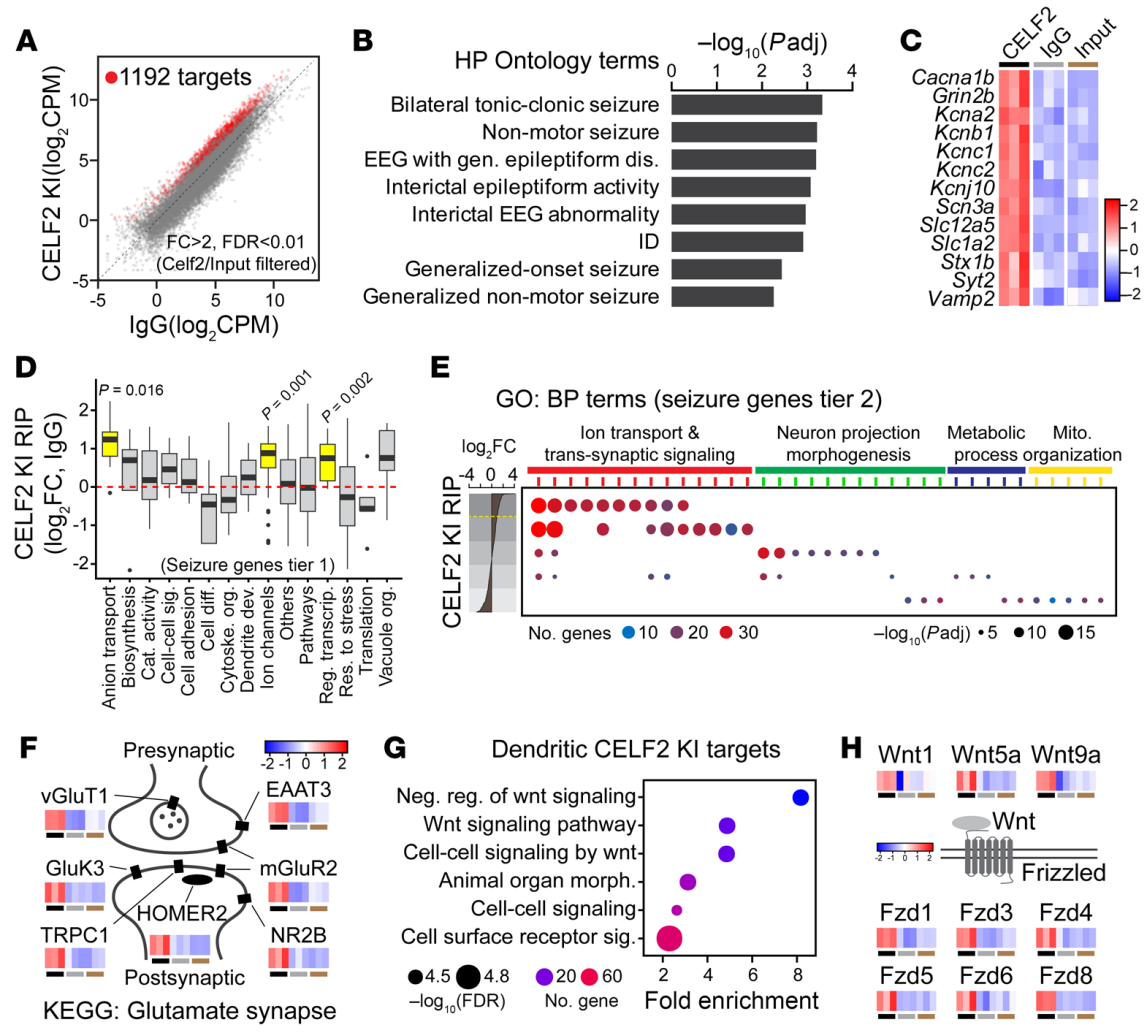
CELF2 mutant in the cytoplasm binds mRNAs of seizure-related genes and regulators of intrinsic excitability. (**A**) Scatterplot showing log_2_-transformed RIP-seq counts per million (CPM) for CELF2 versus IgG. Genes enriched ≥2-fold with FDR < 0.01 relative to IgG RIP and total input are in red. (**B**) Human Phenotype (HP) Ontology analysis of 1,192 CELF2-KI targets, showing top 8 enriched terms with log_10_-transformed adjusted *P* values. (**C**) Heatmap showing *z*-score–transformed expression levels of selected genes from the enriched HP Ontology terms across CELF2-KI RIP, IgG RIP, and total input samples. (**D**) CELF2-KI RIP enrichment (log_2_ fold-change of RIP/IgG) for 142 seizure-related genes (tier 1) (38), across 15 functional groups based on Gene Ontology (GO) terms. Wilcoxon’s signed-rank test; groups with significantly higher enrichment are in yellow. Box plots show the interquartile range (IQR), median (line), and 1.5× IQR (whiskers). (**E**) Dot plot showing enriched GO terms from the analysis of 514 tier 2 seizure-related genes, grouped into 5 equal-sized bins based on their CELF2-KI RIP enrichment, as shown on the left. The yellow dotted line marks a 2-fold change in RIP/IgG. The enriched GO terms are grouped into 4 Biological Process (BP) categories. Color indicates the number of genes mapped to each term, while dot size represents the adjusted *P* value. (**F**) Schematic diagram of glutamate synapse–related genes identified through KEGG analysis of CELF2-KI targets, accompanied by heatmaps showing their *z*-score transformed expression levels across CELF2-KI RIP, IgG RIP, and total input samples as in **C**. (**G**) Dot plot showing the top 6 enriched GO BP terms with fold enrichment values, from the analysis of 185 CELF2-KI target mRNA identified as dendritic mRNA (39). (**H**) Schematic of Wnt ligands and Frizzled receptors identified as CELF2-KI targets, with heatmaps showing their *z*-score expression levels as in **C**. GO, Gene Ontology; KEGG, Kyoto Encyclopedia of Genes and Genomes.

To understand how cytoplasmic accumulation of p.Arg493His mutant induces hyperexcitability relevant to seizures, we sought to identify its target mRNAs in the cytoplasm. We performed RNA immunoprecipitation-sequencing (RIP-seq) with a specific antibody against CELF2 for cytoplasmic RNA isolated from the P0 KI mouse cortex, a time point when CELF2 is normally in the nucleus of WT neurons (Supplemental Figure 5A) (see Methods). Three biological replicates of CELF2-KI RIPs identified 1,192 protein-coding mRNAs as targets enriched by at least 2-fold compared with control IgG RIPs and total input samples, with an FDR < 0.01 (Figure 5A, Supplemental Figure 5B, and Supplemental Table 3). Human Phenotype Ontology analysis of these target mRNAs revealed a specific enrichment of terms related to epilepsy/seizures and ID (Figure 5B and Supplemental Table 4), consistent with observed patient phenotypes. Examples of genes associated with these terms included voltage-gated ion channels (e.g., *Kcna2*, *Scn3a*), ion and glutamate transporters (e.g., *Slc12a5*, *Slc1a2*), and regulators of synaptic transmissions (e.g., *Grin2b*, *Syt2*) (Figure 5C and Supplemental Table 5). Pathogenic variants in these genes are associated not only with epilepsy, seizures, and ID but also with neurodevelopmental disabilities, including those seen in patients with CELF2 variants. For instance, GoF mutations in *SCN3A* are associated with epileptic encephalopathy, ID, and cortical malformation (30, 31), while *SLC12A5* variants are associated with epilepsy of infancy with migrating focal seizures and profound ID and ASD (32–34). Importantly, alterations in the function of these genes have been shown to change neuronal excitability in mouse and human hCNs (35–37), phenocopying KI mice and p.R493H patient hCNs.

To ask which molecular processes may mediate the collective contribution of target mRNAs to neuronal excitability and epilepsy, we examined known epilepsy-associated genes, which cause seizures through different mechanisms (38). We found only a slight, yet significant, enrichment of 143 high-confidence epilepsy genes (tier 1) in CELF2-KI RIPs, compared with randomly selected control genes (Supplemental Figure 5C) (38). This suggests the CELF2 mutant influences some, but not all, seizure-related pathways. Indeed, functional grouping revealed that the enriched genes were specifically associated with ion transport, ion channels, and transcription but not with pathways related to cell differentiation, catalytic activity, and others (Figure 5D and Supplemental Table 6). Further analysis of 514 broader epilepsy genes (tier 2) also showed that the top-enriched genes were primarily involved in ion transport and synaptic regulation (Figure 5E and Supplemental Table 7), consistent with their encoded proteins localizing to synapses, dendrites, and membrane structures (Supplemental Figure 5D). In contrast, other tier 2 genes related to neuron structure and metabolism showed no enrichment in CELF2-KI RIPs (Figure 5E). GO and KEGG analysis of the targets highlighted cell-cell signaling and glutamate synapse pathways (Figure 5F and Supplemental Figure 5E), further supporting a direct link of CELF2 to excitatory synaptic signaling. Interestingly, CELF2 has been shown to bind mRNAs regulating neuronal excitability in human cortical organoids (9). Despite model differences, we found approximately 10% overlap in target mRNAs of the CELF2 mutant, linked to the dendritic compartment and glutamatergic synapses (Supplemental Figure 5, F and G, and Supplemental Table 8).

This compartmentalization feature of target mRNAs suggests that activity-dependent changes in CELF2’s cytoplasmic availability may influence local mRNA regulation in dendrites to modulate synaptic functions and neuronal excitability. To explore this possibility, we examined dendritic mRNAs identified in hippocampal CA1 neurons (39). Approximately 15% of target mRNAs were found in dendrites, consistent with GO analysis showing their enrichment in synapses, cell-cell junctions, and somatodendritic compartments, with roles in cell-cell and receptor signaling (Figure 5G, Supplemental Figure 5H, and Supplemental Table 9). Notably, these included regulators of Wnt signaling pathways, such as Wnt ligands and receptors (Figure 5H). Both canonical and noncanonical Wnt signaling are known to regulate intrinsic firing properties, with their dysregulation linked to epilepsy (40, 41) and ID (42–44). WNT5A, for instance, can act as an autocrine factor that rapidly modulates synaptic transmission and intrinsic excitability by remodeling spines (45) and mobilizing glutamate receptors (41). These rapid effects of WNT5A align with its expression and secretion under the control of local translation in response to synaptic activity (46–48). Accordingly, disruption of WNT5A expression and function has been shown to impair learning and memory in mice (49).

### CELF2 mislocalization increases seizure susceptibility and perturbs learning and memory processes in mice.

**Figure 6:**
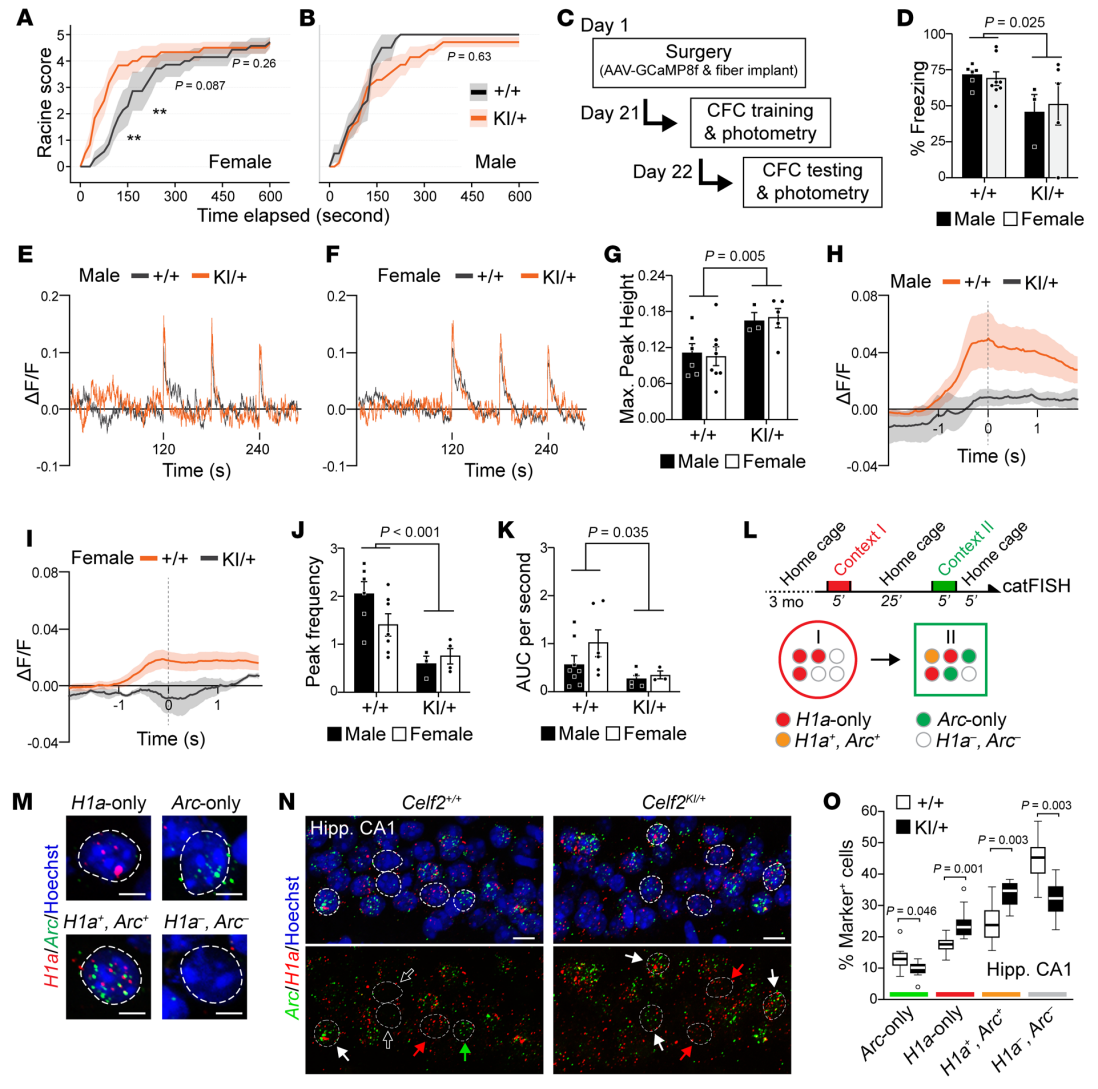
Neuronal hyperactivity caused by CELF2 mislocalization elevates seizure susceptibility and disrupts learning and memory. (**A** and **B**) Racine score progression following PTZ injection in female (**A**) and male (**B**) WT and KI mice. Shaded areas represent SEM. Mantel-Cox log-rank test with Benjamini-Hochberg correction. **P = 0.008. (**C**) Schematic of the experimental setup for fiber photometry and contextual fear conditioning (CFC). Three weeks after AAV-mediated GCaMP8f expression and fiber implantation in the CA1 region, neuronal responses were recorded during foot shock trials and subsequent retrieval tests. (**D**) Contextual fear memory retention in mice. (**E** and **F**) The fractional change in fluorescence relative to a baseline signal (ΔF/F) traces recorded during CFC acquisition. (**G**) Quantification of photometry amplitude in response to foot shocks. (**H** and **I**) Photometry ΔF/F traces during memory retrieval, aligned to freezing-to-moving transitions (±2 seconds from freezing offset). (**J** and **K**) Quantification of peak frequency (**J**) and AUC (**K**) of the photometry signal during freezing-to-moving transitions. (**L**) Schematic showing sequential context exploration and catFISH with 4 nuclear expression profiles of immediate-early genes (IEGs) *Arc* and *H1a* mRNA in response to contexts I and II. (**M**) Confocal images of *H1a* (red) and *Arc* (green) mRNA in hippocampal CA1 neurons. (**N**) Confocal images of hippocampal CA1 regions following catFISH after context exploration. Red, green, white, and empty arrows indicate *H1a*-only, *Arc*-only, double-positive, and IEG-negative neurons, respectively. Nuclei were counterstained with Hoechst 33258 and outlined with dashed white lines. (**O**) Quantification of IEG expression in CA1 neurons from **N**. *n* ≥ 9 each. Means ± SEM. Each dot represents 1 animal. Two-way ANOVA, Tukey’s post hoc test (**D**, **G**, **J**, and **K**), and Mann-Whitney *U* test (**O**). Scale bars: 5 μm (**M**), 10 μm (**N**).

We next asked whether KI mice exhibit reduced seizure threshold. Given that seizure induction is highly paradigm dependent, with different protocols engaging distinct molecular and circuit mechanisms, and that our RIP and MEA analyses implicated synaptic network dysregulation rather than all seizure-related pathways, we employed 3 complementary protocols, including the pentylenetetrazole (PTZ) clonic seizure threshold test, repeated low-dose kainic acid (KA) injection, and hyperthermia-induced seizures. Following a single PTZ injection, both WT and KI mice exhibited stereotyped seizure progression, as reflected by incrementally increasing Racine scores over time (Supplemental Figure 6A). Notably, female KI mice exhibited a significantly shorter latency to seizure onset and faster progression to Racine score 2, with a trend toward earlier clonic convulsions, which indicates a reduction in seizure threshold (Figure 6, A and B). In contrast, this reduction was not observed in the repeated KA injection and hyperthermia paradigms (Supplemental Figure 6, B–D), suggesting that CELF2-associated neuronal hyperexcitability affects seizure-relevant circuits through selective mechanisms, rather than producing a nonspecific disruption of global neuronal activity.

Activity-dependent plasticity of intrinsic excitability is also critical for the formation of neuronal ensembles underlying learning and memory (19). The ID and speech delay observed in patients prompted us to investigate whether impaired activity-dependent CELF2 translocation disrupts neuronal ensemble activation patterns critical for learning and memory. To this end, AAV9-GCaMP8f was injected into hippocampal CA1 of WT and KI mice to record Ca^2+^ activity as a proxy for population neuronal activity via fiber photometry during contextual fear conditioning (Figure 6C). KI mice of both sexes showed impaired contextual fear memory retention at 24 hours (Figure 6D). During the acquisition phase, KI mice showed significantly larger population activity in response to foot shocks compared with WT littermates (Figure 6, E–G), consistent with the hyperexcitability of KI neurons. Interestingly, despite the hyperactivity during acquisition, CA1 population responses were substantially reduced during the retrieval test (Figure 6, H and I), potentially reflecting the impaired retrieval. During movement bouts in the retrieval session, we observed decreases in the peak frequency and AUC of the photometry signal in both sexes, with no significant changes observed during bouts of freezing (Figure 6, J and K, and Supplemental Figure 6, E–H).

Neurons with higher intrinsic excitability are preferentially recruited during memory encoding, while subsequent excitability reduction in previously activated neuron ensembles enables memory separation (19). Persistent hyperexcitability disrupting this dynamic has been shown to cause memory impairment (50). Consistent with this, KI mice showed a significant reduction in freezing responses during fear memory retrieval (Figure 6D).

To determine whether impaired freezing responses reflects aberrant recruitment and ensemble separation, we used cellular compartment analysis of temporal activity by fluorescent in situ hybridization (catFISH) to label CA1 neuronal populations activated during sequential exploration of 2 distinct contexts (Figure 6L and Supplemental Figure 6I) (51). This approach exploits the differential temporal dynamics of IEGs *Arc* (activated within 5 minutes and rapidly translocated from the nucleus) and *Homer1a* (*H1a*; activated at 30 minutes) to precisely label context-specific neuronal ensembles. As expected, in the nucleus, we found CA1 neurons with only *H1a* mRNA (earlier activation by context I), *Arc* mRNA (recent activation by context II), both mRNAs (activation by both contexts), and no IEG mRNA (no activation) (Figure 6M). KI mice showed significantly more *Arc*- and *H1a*-positive CA1 neurons than WT (Figure 6, N and O), indicating increased recruitment of neurons in response to context exploration. Notably, while total *Arc*-positive neurons were elevated in KI mice responding to context II, neurons expressing only *Arc* were reduced, with a corresponding increase in *Arc*/*H1a* double-positive neurons, suggesting aberrant reactivation of context I ensembles during context II exploration (Supplemental Figure 6I). This overlap was specific to CA1 and absent in CA3 and the anterior cingulate cortex (Supplemental Figure 6, J and K), consistent with CA1’s role in context memory formation and retrieval (52). It is likely that other tasks that differentially engage distinct brain regions would also display enhanced excitability in KI mice.

### Drug screening reveals AKT signaling as a regulator of CELF2 translocation and a potential target for restoring neuronal hyperactivity.

**Figure 7:**
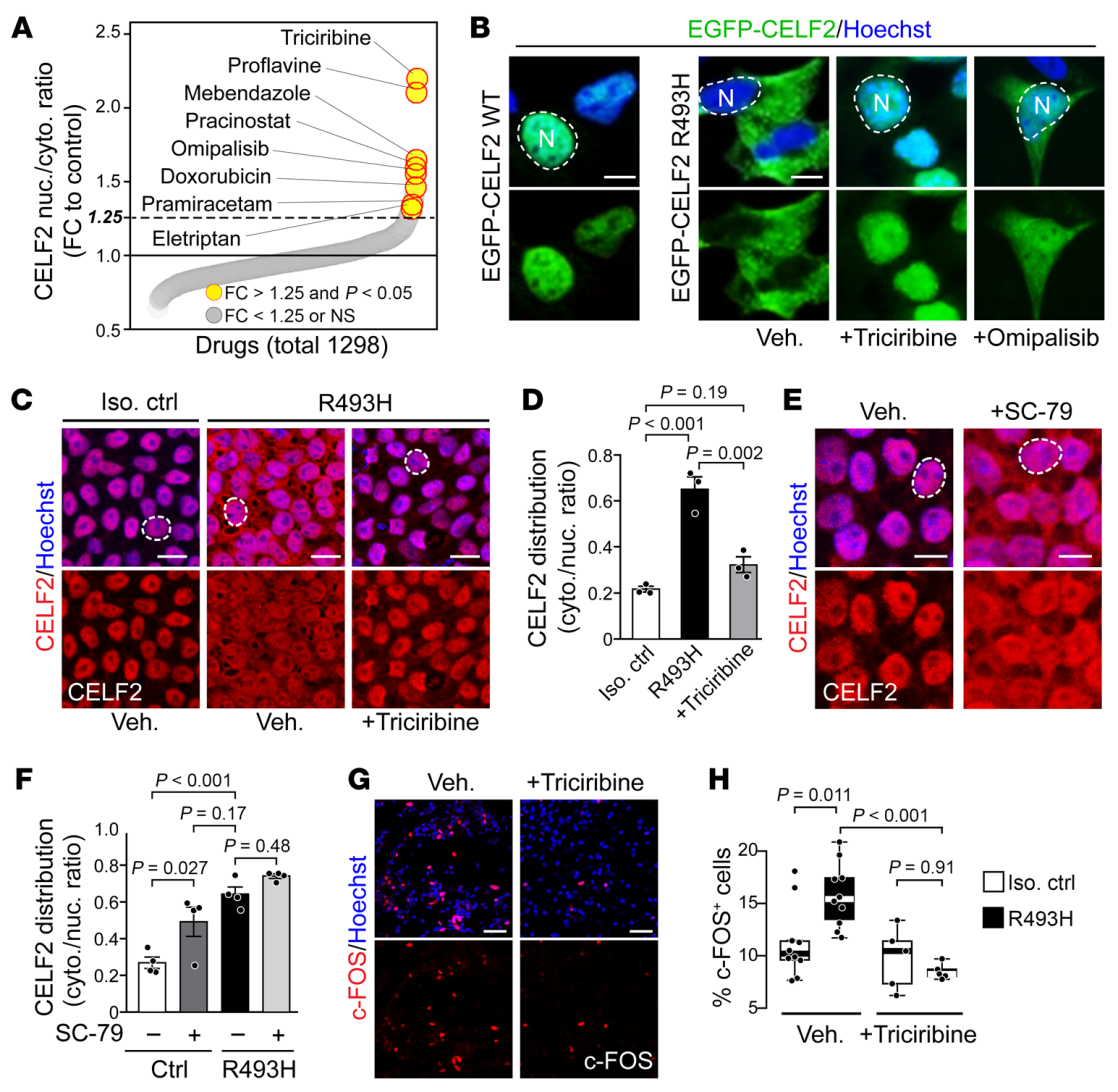
High-throughput drug screening identifies AKT signaling as a regulator of CELF2 translocation and a target for rescuing hCN hyperactivity. (**A**) Drug effect plot showing compounds ranked by their effectiveness in restoring nuclear localization of EGFP-CELF2 p.Arg493His in HEK293 cells, compared with DMSO control. Top-ranked compounds are in yellow with red circles. (**B**) Confocal images of HEK293 cells expressing EGFP-CELF2 WT or p.Arg493His (both green), treated with vehicle, triciribine, or omipalisib. (**C** and **D**) Confocal images of isogenic control or p.Arg493His hiPSCs treated with vehicle or triciribine (1 μM, 24 hours), immunostained for CELF2 (red) (**C**), and quantifications of CELF2 distribution (**D**). *n* = 3 (53 cells each). (**E** and **F**) Confocal images of control hiPSCs treated with vehicle or SC-79, immunostained for CELF2 (red) (**E**) and quantifications of CELF2 distribution (**F**). *n* = 4 (≥100 cells each). (**G** and **H**) Confocal images of p.Arg493His hCNs treated with vehicle or triciribine, immunostained for c-FOS (red) (**G**), and quantifications of c-FOS^+^ cells in isogenic control and p.Arg493His hCNs (**H**). *n* ≥ 5 (≥1,000 cells each). Box plots show the interquartile range (IQR), median (line), and 1.5× IQR (whiskers). Nuclei were counterstained with Hoechst 33258 (blue, **B**, **C**, and **E**) and outlined with dashed white lines, with “N” denoting the nucleus. Means ± SEM. Each dot represents 1 experiment. One-way ANOVA, Tukey’s post hoc test (**D**), 2-way ANOVA with Tukey’s post hoc test (**F** and **H**). Scale bars: 5 μm (**B**), 10 μm (**C** and **E**), 50 μm (**G**).

CELF2 contains multiple NLSs and nuclear export signals that collectively determine its localization (3). To ask whether the mislocalization of mutant CELF2 is reversible, we generated a stable HEK293 line expressing EGFP-tagged p.Arg493His and performed high-throughput screening of approximately 1,300 small molecule compounds (1 μM, 24 hours) from an FDA-approved Drug Library (Selleckchem). Immunostaining and subcellular fractionation confirmed cytoplasmic accumulation of p.Arg493His CELF2, in contrast with the predominant nuclear localization of WT CELF2 in stable cell lines (Supplemental Figure 7, A–C). After 2 rounds of screening, we identified 8 compounds that significantly restored nuclear p.Arg493His CELF2 (Figure 7A, Supplemental Figure 7D, and Supplemental Table 10). Triciribine and omipalisib, which both target the PI3K/AKT pathway, were among the most effective hits. Notably, triciribine, a selective AKT inhibitor, fully restored mutant CELF2 nuclear localization to WT levels (Figure 7B). Treatment of p.Arg493His patient-derived hiPSCs with triciribine similarly led to robust nuclear restoration of mutant CELF2 proteins, comparable to isogenic controls (Figure 7, C and D). Conversely, treatment with the pan-AKT activator SC-79 induced significant cytoplasmic translocation of WT CELF2 and further enhanced the cytoplasmic accumulation of the mutant (Figure 7, E and F). These results suggest that CELF2 nucleocytoplasmic shuttling is regulated by AKT-mediated signaling.

Given the robust nuclear restoration of mutant CELF2 by AKT inhibition, we next asked whether this could also rescue the associated hyperactivity. To test this, we treated hiPSC-derived hCNs with triciribine and observed a significant reduction in the c-FOS^+^ p.Arg493His cells to a comparable level as isogenic control hCNs (Figure 7, G and H). This suggests that CELF2 mislocalization–induced neuronal hyperactivity is primarily a result of impaired electrical properties rather than growth defects, consistent with our findings on CELF2 target mRNAs.

## Discussion

Dysregulation of RBPs contributes to a broad spectrum of human disorders, yet their pleiotropic roles and complex regulation make it challenging to determine how specific genetic variants drive disease pathogenesis, particularly in rare diseases with high clinical and genetic heterogeneity. In this study, we focus on *CELF2* variants and provide converging clinical and mechanistic evidence that CELF2 mislocalization underlies neuronal hyperactivity, seizures, and learning deficits. First, clinical characterization of 18 individuals suggests a potential association of mislocalization variants, but not protein-null variants, with epileptic seizures. Second, *Celf2*-KI mice carrying a patient mislocalization variant, but not *Celf2^+/–^* mice, exhibited neuronal hyperactivity phenocopying patient-derived neurons. This was accompanied by network hyperactivity, elevated seizure susceptibility, and learning and memory deficits in KI mice, in line with the clinical presentation of affected individuals. Third, mislocalized CELF2-mutant protein aberrantly binds mRNAs encoding ion channels and synaptic signaling modulators associated with epileptic encephalopathy. Finally, CELF2 nucleocytoplasmic shuttling is activity dependent and regulated by AKT signaling. AKT inhibition restores nuclear CELF2 localization and rescues hyperactivity in patient-derived neurons.

All reported CELF2 variants to date are heterozygous (7, 10), consistent with a dominant model (i.e., haploinsufficiency, GoF, dominant negative). Our findings suggest that these variants likely act through diverse mechanisms, differentially affecting CELF2 localization, expression, or activity and thereby contributing to varied clinical phenotypes. Supporting this, several nonsense and frameshift variants predicted to trigger NMD showed cell type–specific effects; while both p.Gln230Ter and p.Gln34Ter induced NMD in hiPSCs, p.Gln34Ter escaped NMD in HEK293 cells (Figure 1, C and D), consistent with known variation in NMD efficiency across cell type, developmental stages, and premature termination codon position (53, 54). Whether these findings reflect endogenous protein behavior in patients remains to be determined. Nonetheless, it is tempting to speculate that p.Gln34Ter and potentially other PTVs may produce truncated CELF2 lacking different functional domains or act as an RNA decoy, disrupting normal CELF2 activity or target RNA regulation in a cell type–specific manner.

On the other hand, our data provide evidence that mislocalization variants exert effects beyond mere loss of nuclear CELF2 function, consistent with the more severe clinical features observed in affected individuals, including epileptic seizures and structural brain abnormalities (Supplemental Tables 1 and 2) (7, 10). A parallel can be drawn with amyotrophic lateral sclerosis, where mislocalization of the disease-causing RBP TDP-43 results in cytoplasmic GoF acting concurrently with nuclear LoF to drive pathogenesis (55, 56). Similar dual pathomechanisms are also seen in other RBPs like FUS (57, 58) and hnRNPA2 (59). Moreover, cytoplasmic FUS accumulation, rather than nuclear depletion, can selectively induce neuronal defects and social behavioral abnormalities (60), highlighting compartment-specific impacts. Likewise, our findings indicate that neuronal hyperactivity and the related pathological outcomes are likely driven by cytoplasmic GoF mechanisms involving mislocalized CELF2. This is supported by the observation that neuronal hyperactivity was present in KI mice carrying the p.Arg493His mislocalization variant but absent in *Celf2*^+/–^ mice (Figure 3, D–M), consistent with the observations in patients reported to date (Figure 1B). Although p.Arg493His resides within RRM3, our previous findings suggest that the mutant protein retains RNA-binding ability, driving phenotypic changes comparable to those seen with WT CELF2 (7). We also noticed that a frameshift variant (p.Gly414AlafsTer45) that escapes NMD but produces a truncated CELF2 lacking the entire RRM3 domain induces mislocalization yet is not associated with seizures (Figure 1, E–G). In contrast, the nonsense variant p.Tyr508Ter (10), which removes only the last amino acid and preserves RRM3, and the missense variants p.Gly268Cys and p.Asn364Ile, located in the divergent domain upstream of RRM3, all cause CELF2 mislocalization and are associated with seizures, resembling the effects of NLS-disrupting missense variants. Together, these observations support a cytoplasmic GoF mechanism that requires RRM3 integrity. Nevertheless, this interpretation is based on a limited number of variants identified so far, and validation in larger cohorts with a broader spectrum of CELF2 variants will be essential to test this model.

The potential requirement for CELF2 RNA-binding activity raises the possibility that cytoplasmic retention of mutant CELF2 drives seizure susceptibility by disrupting specific neuronal and network pathways. Supporting this, mislocalized CELF2-mutant protein preferentially binds seizure-associated mRNAs involved in signal transmission and ion transport, but not those linked to metabolic or other pathways, highlighting its selective impact on neuronal excitability and network activity (Figure 5, C–E). Disrupting the expression of these genes, such as the glutamate transporter *VGLUT1* (61, 62) and potassium channels *KCNA2* (63) and *KCNB1* (64), induces neuronal hyperactivity, mirroring the phenotypes observed in KI mice. Many mRNAs associated with synaptic function undergo local translation within dendritic compartments to facilitate rapid and localized modulation of neuronal activity (65). We found several target mRNAs localized in dendrites, including those encoding Wnt ligands and receptors. These mRNAs have been shown to be extensively regulated at the translational level (47, 66) and exhibit expression and localization changes in response to neuronal activity (46, 67–69). This activity-dependent regulation enables rapid modulation of Wnt signaling to influence synaptic function and neuronal excitability (41, 68, 70–73), and its dysregulation has been linked to epilepsy (40, 74). This target selectivity of mutant CELF2 may also contribute to the differential responses observed across seizure induction paradigms in KI mice (Figure 6A and Supplemental Figure 6, B and D). Although the mechanisms underlying the elevated sensitivity of KI mice to GABAergic disinhibition remain to be determined, this selective vulnerability suggests that inhibitory signaling and network stability may represent tractable therapeutic targets in CELF2-associated seizure conditions. It remains possible that CELF2 mislocalization–induced hyperactivity reflects accelerated neuronal maturation, since CELF2 translocates in mouse neurons around the onset of firing. However, as hyperactivity persists in neurons at a more mature stage and is reversible in hCNs, our data favor a direct role in regulating neuronal activity and warrant future studies of its impact on neuronal maturation.

An interesting finding of this study is that CELF2 undergoes dynamic shuttling in neurons, with neuronal activation driving its translocation from the cytoplasm into the nucleus. This nucleocytoplasmic shuttling appears to be a general mechanism regulating CELF2 function. For example, cytoplasmic CELF2 translocates into the nucleus to drive neurogenic differentiation in embryonic mouse NPCs (7), and a similar transition was seen during chicken heart development (12). Conversely, γ-irradiation induces CELF2 nuclear-to-cytoplasmic translocation in human cancer cells (75). Although activity-dependent shuttling of RBPs and their regulatory roles in neurons remain underexplored, a few earlier studies provide initial insights. For example, it has been shown that neuronal activation promotes the cytoplasmic translocation of nuclear NOVA, an epilepsy-associated RBP, which allows it to change synaptic protein production and consequently neuronal activity (76–78). Similarly, neuronal activation triggers the rapid trafficking of 2 other RBPs, ZBP1 and SAM68, from the cell body into dendrites and spines, allowing local translation of specific synaptic mRNAs (79, 80).

Given this, our findings support a model in which CELF2 shuttling facilitates activity-dependent modulation of excitability, which is critical for learning and memory. In this regard, fluctuations in intrinsic excitability may guide the formation of memory-encoding neuronal ensembles, with more excitable neurons preferentially activated to store specific memories (19). Subsequent homeostatic mechanisms lower their excitability, allowing other unallocated neurons with increased excitability to encode new memories (81). Supporting our model, KI mice showed a greater CA1 population response during the acquisition phase of a CFC task (Figure 6, E–G). Interestingly, KI mice demonstrated impaired retention of fear memory 24 hours later and significantly reduced CA1 activity during the memory retrieval session (Figure 6, D and H–K), consistent with increased recruitment of neurons because of their aberrantly elevated excitability. The disruption of activity-driven CELF2 nuclear translocation may also impair homeostatic excitability reduction in these neurons. As a result, KI mice showed a significantly greater overlap of neurons activated by exploring distinct contexts (Figure 6O). Conversely, *Celf2^+/–^* mice exhibit normal learning and memory but show social deficits (8), further indicating the divergent pathomechanisms contributing to phenotypic heterogeneity in these patients. In light of the existence of multiple CELF family members, they are likely to act differentially to regulate neuronal functions, as LoF mutations in *CELF4* are associated with seizures (82–84), unlike in *CELF2*. These differences underscore the need for tailored patient management and variant-specific therapeutic approaches. These observations also carry implications for syndrome nomenclature. At present, OMIM associates pathogenic variants in *CELF2* with Developmental and Epileptic Encephalopathy 97 (DEE 97, 619561), although it is plausible that at least 2 distinct NDDs may arise from heterozygous pathogenic variation in *CELF2*. Using the dyadic approach to syndrome delineation (85), we could tentatively consider a CELF2-LoF NDD and a CELF2-related DEE. Identification of additional variants with differential impacts on CELF2 functions will be essential to substantiate this framework.

What mechanisms regulate CELF2 localization and enable its dynamic shuttling in response to neuronal activity? Deletion of C-terminal NLSs causes cytoplasmic localization, but this effect can be reversed by simultaneously removing N-terminal nuclear export signal motifs (3), suggesting that CELF2 localization is determined by the combinatorial actions of opposing nuclear import and export processes, potentially under the control of specific cellular signaling mechanisms. Our small molecule screening and other evidence implicate AKT signaling in this regulation. We found that PI3K/AKT inhibition induced nuclear translocation of mutant CELF2, whereas AKT activation promoted its cytoplasmic localization (Figure 7, D and F). Activating mutations in AKT signaling are associated with cortical malformations and epilepsy (86–88), which partially overlap with clinical features in patients with CELF2 mislocalization. AKT inhibition, on the other hand, suppresses neuronal hyperactivity (89), aligning with its rescue of hyperactivity defects in patient-derived hCNs (Figure 7H). Notably, direct AKT inhibition has shown antiseizure and antiepileptogenic effects in animal models (90, 91), and indirect modulation of AKT signaling has demonstrated clinical benefit in some forms of epilepsy (92, 93). However, several considerations will need to be addressed to translate the therapeutic potential of AKT inhibition for managing CELF2-related conditions. First, preclinical validation in *Celf2*-KI mice will be required to establish whether AKT inhibition reduces seizure susceptibility in vivo. Second, whether AKT inhibition can restore normal CELF2 localization across all pathogenic variants remains unknown. Assessing the converging and diverging effects will be valuable for the delineation of variant-specific mechanisms. Third, given that AKT plays pleiotropic roles in cell survival, growth, and other physiological processes, chronic inhibition raises concerns about systemic toxicity. A precise mechanistic understanding of how AKT controls CELF2 translocation will be critical for defining the optimal intervention timing and for developing strategies with greater precision to prevent or slow epilepsy progression, for example, by directly modulating AKT-dependent phosphorylation on CELF2.

Indeed, CELF2 has multiple predicted AKT phosphorylation sites (94). Notably, Ser28 in CELF1, corresponding to Ser52 in CELF2, is a confirmed AKT target (95). Phosphorylation at this site drives CELF1 cytoplasmic translocation, while blocking its phosphorylation results in nuclear localization (95), which is consistent with our findings, although none of the reported CELF2 mislocalization variants are proximal to Ser52 in either the primary sequence or AlphaFold-predicted 3D structure (AF-O95319-4-F1-v6). Phosphorylation has been shown to regulate CELF family members in diverse ways, depending on the sites and upstream kinases involved. For example, in type 1 myotonic dystrophy (DM1), PKC-mediated hyperphosphorylation stabilizes CELF1 protein (96), while CDK4/6-mediated phosphorylation of Ser302 in CELF1 modulates its interaction with EIF2A and mRNA targets and its subcellular localization (95, 97). In DM1 patient-derived cortical organoids, hyperphosphorylation of CELF2 disrupts its interaction with mRNAs encoding synaptic regulators, causing hyperexcitability of glutamatergic neurons (9). Despite the increased activity, CELF2 does not show a shift in localization within these neurons (9), raising the possibility that phosphorylation may promote its cytoplasmic retention, potentially contributing to the hyperexcitability phenotype. Nonetheless, it is also plausible that AKT does not directly phosphorylate CELF2 but instead acts via indirect pathways. Future studies identifying specific phosphorylation sites on CELF2 or other indirect pathways will help reveal these mechanisms governing CELF2 shuttling and their impact on neuronal activity.

## Methods

### Sex as a biological variable.

Sex was considered a biological variable for some experiments. Clinical cohort patients included both male and female individuals. Patient-derived cells were obtained from a female donor and the control from a male donor. For animal experiments, both male and female mice were used throughout.

### Patients.

Individuals with *CELF2* variants were identified through trio exome sequencing and evaluated by clinical geneticists. Family history and clinical features were obtained by the physician at each site and are summarized in Supplemental Tables 1 and 2.

### Animals.

CD1 and C57BL/6 (Charles River Laboratories) mice were used for immunohistochemistry and primary culture. The morning of plug detection was designated as E0.5. Heterozygous *Celf2*-KI mice harboring the p.Arg493His variant were generated using CRISPR/Cas9-mediated genome editing at the University of Calgary Transgenic Facility and validated by Sanger sequencing. *Celf2*-knockout mice were generated by crossing CMV-Cre deleter mice with conditional knockout mice (*Celf2^+/fl^*) (EMMA: 11977) to achieve CMV-Cre–mediated deletion of *loxP*-flanked exon 2. Resulting lines were backcrossed for 5 generations prior to use.

### hiPSC culture and neuronal differentiation.

hiPSCs were maintained on Matrigel-coated plates in mTeSR1 or mTeSR Plus (STEMCELL Technologies). hNPCs were generated using the STEMdiff SMADi Neural Induction Kit (STEMCELL Technologies) and maintained in KnockOut DMEM-F12 (Thermo Fisher Scientific) supplemented with N2 (1:100), B27 (1:50), EGF (20 ng/mL), FGF2 (20 ng/mL), and laminin (1 μg/mL). hNPCs were differentiated into neurons in BrainPhys Medium (STEMCELL Technologies) supplemented with N2 (1:100), B27 (1:50), BDNF (20 ng/mL), GDNF (20 ng/mL), and laminin (1 μg/mL).

### Statistics.

All data are presented as mean ± SEM. Statistical analyses were performed using R, Microsoft Excel, or GraphPad Prism, with specific statistical tests detailed in the figure legends. A *P* value of less than 0.05 was considered statistically significant.

### Study approval.

Verbal or written consent was obtained from study participants or their parents prior to participation. Patient-derived cell studies were approved by the Conjoint Health Research Ethics Board at the University of Calgary (REB22-1105). Collection and processing of human fetal tissues were approved by Chongqing Medical University and conducted in accordance with institutional guidelines. Animal use was approved by the Animal Care Committee at the University of Calgary (AC25-0053, AC24-0082, AC25-0005-7) and conducted in accordance with the Canadian Council of Animal Care.

### Data availability.

RNA-sequencing data generated in this study are publicly available through the NCBI Gene Expression Omnibus under the accession number GSE325228. Values for all data points in graphs are reported in the Supporting Data Values file. Additional details can be found in Supplemental Methods. Antibodies and primer sequences used in this study are provided in Supplemental Tables 11 and 12, respectively.

## Author contributions

GY and AMI contributed to conceptualization. GY and AMI contributed to conceptualization. GY provided project supervision. MJM, CQ, BK, SS, GV, AB, AV, ZS, RJL, GM, FTMT, MT, CP, PL, MMM, DRL, RCC, YI, EP, AL, ET, DS, ES, JC, MD, DC, BI, BC, and AMI performed clinical assessment and human phenotyping. MH, MRA, YR, SVS, YY, YYO, MN, RDK, DF, LW, CJG, GW, MK, and BR conducted investigation. MH, MRA, YR, SVS, YY, YYO, MN, RDK, LW, GW, IC, DK, GCT, JE, GH, SDR, DJM, AMI, JRE, and GY performed data analysis. GY, AMI, JRE, JE, and MJM acquired funding. GY, AMI, and JRE wrote the original draft with input from MH, MRA, and MJM. All authors contributed to manuscript revision and review. The order of authorship among the co–first authors was determined by mutual agreement.

## Conflict of interest

The authors have declared that no conflict of interest exists.

## Funding support

This work is the result of NIH funding and is subject to the NIH Public Access Policy. Through acceptance of this federal funding, the NIH has been given a right to make the work publicly available in PubMed Central.

- Azrieli Foundation “RNA and the Brain” Grant G-2101-15624 (GY and JE).
- CIHR Project Grants 518557 & 512979 (GY, JRE, AMI, MM)
- One Child Every Child Strategic Catalyst Award (GY, AMI, MM).
- Canadian Gene Cure Advanced Therapies for Rare Diseases (Can-GARD) (GY).
- Owerko Centre Small Equipment Grant (GY, DMK).
- Clinical Sequencing Evidence-Generating Research Consortium grants NHGRI U01HG007301 and NICHD 1R01HD112437 (DRL).
- ACHRI Graduate Scholarship (MH).
- CIHR Postdoctoral Fellowship (MRA).
- CSM Postdoctoral Fellowship (MRA).
- ACHRI Postdoctoral Fellowship (MRA).
- Canada Research Chair, Tier II, in Gene Regulation in Brain Development (GY).

## Supplementary Material

Supplemental data

Unedited blot and gel images

Supplemental tables 1-13

Supporting data values

## Version 1 06/11/2026

In-Press Preview

## Version 2 07/15/2026

Electronic publication
