# Supplementary material for: Pathological disruption of CELF2 shuttling causes neuronal hyperactivity, learning deficits, and seizures
Source: J Clin Invest. 2026 Jun 11;136(14):e199698. doi: 10.1172/JCI199698 (PMC13367967; doi:10.1172/JCI199698)
Supplement: Supplemental data [file jci-136-199698-s126.pdf]

Supplementary Materials

This document contains Supplementary Figures 1-7 and Supplemental Methods. Supplementary Tables 1-12 are provided as a separate Excel spreadsheet.

Supplemental Figure 1. Epileptic seizure manifestation in individuals with *CELF2* variants.

Supplemental Figure 2. The p.Arg493His variant changes CELF2 subcellular localization and hCN activity.

Supplemental Figure 3. Mislocalization of CELF2 rather than its LoF causes neuronal hyperactivity in mice.

Supplemental Figure 4. CELF2 shows distinct subcellular localization patterns in the embryonic and adult human brains.

Supplemental Figure 5. RNA-immunoprecipitation-sequencing (RIP-seq) identifies target mRNAs bound by CELF2 p.Arg493His mutant in the cytoplasm.

Supplemental Figure 6. Assessment of seizure susceptibility, neuronal activity, and ensemble dynamics in KI mice.

Supplemental Figure 7. Cell-based screening identifies compounds that modulate the localization of EGFP-tagged CELF2 p.Arg493His.

Supplemental Methods

Supplemental Figures

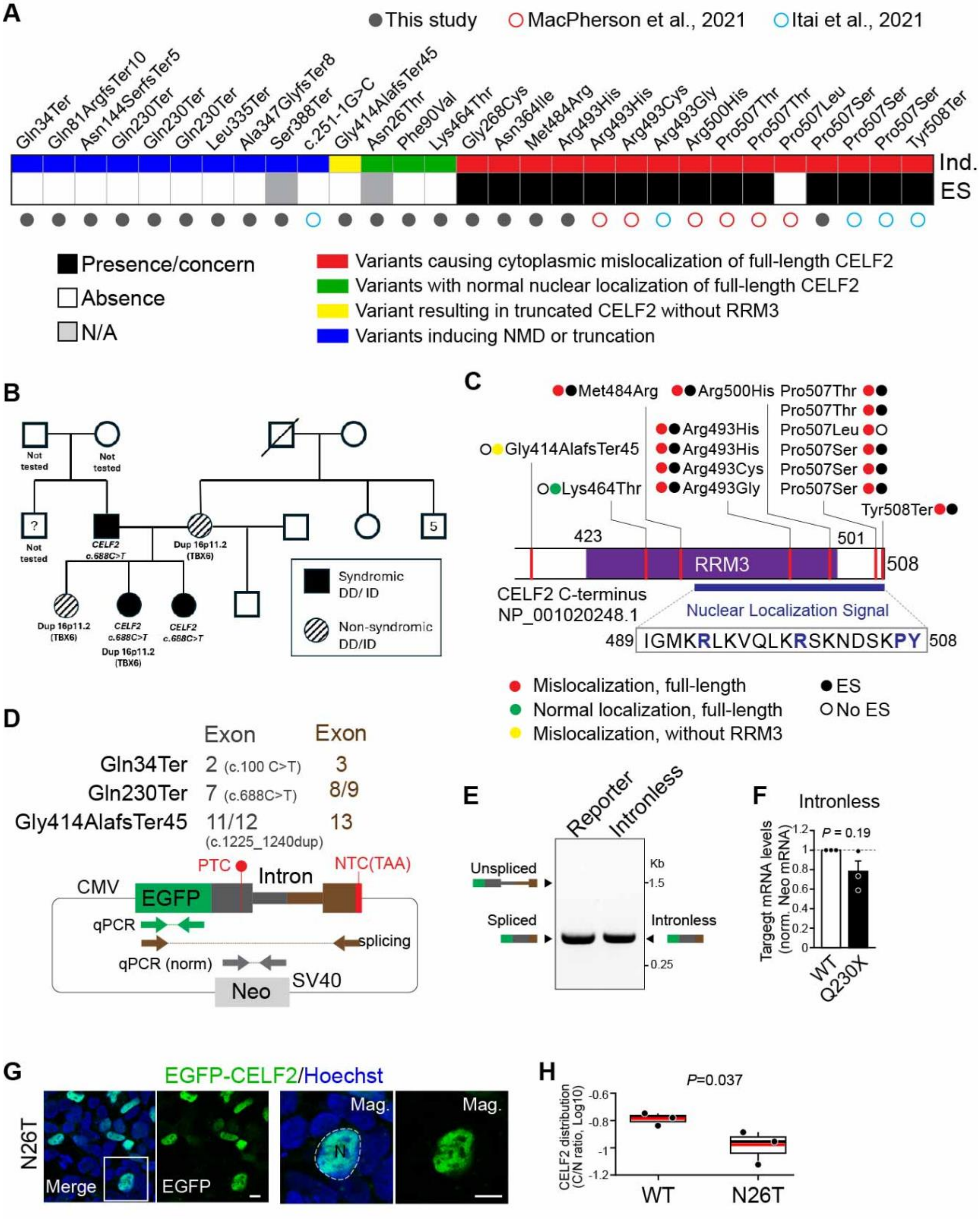

Supplemental Figure 1. Epileptic seizure manifestation in individuals with CELF2 variants. (A) Heatmap of identified variants (this study)(7, 10), and the presence of epileptic seizures (ES) in corresponding individuals, indicated by a black box. A grey box denotes unavailable information**. (B)** Pedigree diagram of a family segregating the c.688C>T, p.Gln230Ter variant. **(C)** Schematic showing the positions of identified variants in the C-terminal region of the CELF2 protein. The RNA-recognition motif 3 (RRM3) overlaps with the nuclear localization signal (NLS), the sequence of which is shown below. Colored dots indicate the effect of each variant on CELF2 subcellular localization and RRM3 integrity, while black circles denote seizure occurrence in the corresponding individuals. **(D)** Schematic of the minigene splicing reporter construct, in which the affected exon is fused in-frame downstream of EGFP, followed by the downstream exon harboring a normal termination codon (NTC), with flanking intronic fragments (see Methods). Variant-induced premature termination codons (PTCs) are indicated within the upstream exon. Primers used for qPCR and splicing assessment are denoted in green, grey, and brown, respectively. **(E)** RT-PCR analysis of splicing efficiency in transfected HEK293 cells using the minigene reporter containing WT Q230 exonic and intronic sequences. An intronless control reporter, comprising the same exons but lacking intronic fragments, was included as a reference. **(F)** qPCR analysis of intronless reporters containing either WT exons or exons harboring the Q230X variant, expressed in HEK293 cells. Data are presented as means ± SEM, normalized to WT. Each dot represents one experiment. One-sample t-test. **(G)** Confocal images of HEK293 cells expressing WT EGFP-CELF2 or the p.N26T variant (green). White box areas are shown at higher magnification to the right. Nuclei were counterstained with Hoechst 33258 (blue) and are outlined with dashed white lines. 'N' denotes the nucleus. **(H)** Quantifications of the cytoplasmic-to-nuclear ratio of CELF2 levels, from (E). *n* = 5 (60 cells each). Unpaired t-test. Scale bars, 5 μm.

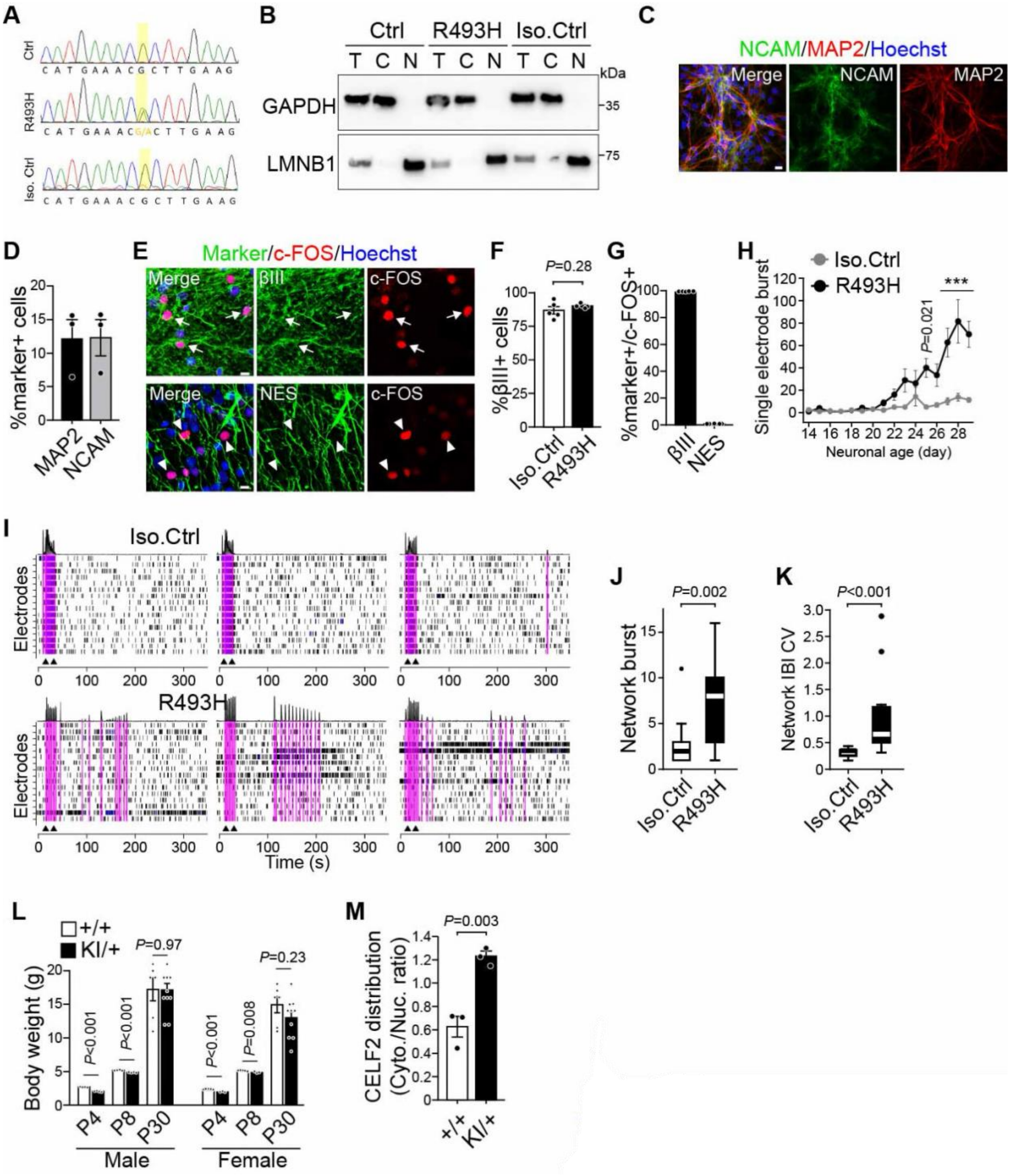

Supplemental Figure 2. The p.Arg493His variant changes CELF2 subcellular localization and hCN activity. (A) Sanger sequencing confirming the heterozygous G>A (*CELF2*, p.R493H) mutation in proband-derived hiPSCs and its correction in isogenic control (Iso.Ctrl) hiPSCs. **(B)** Western blots of total lysates (T), cytoplasmic (C), and nuclear (N) fractions from control, p.R493H, and Iso.Ctrl hiPSCs, probed for

GAPDH (cytoplasmic marker) and LMNB1 (nuclear marker). **(C-G)** Analysis of hCN cultures 20 days post neural differentiation, immunostained for the indicated markers, showing the expression of NCAM (green) and MAP2 (red) (C) and NESTIN (NES) or βIII-tubulin (βIII) (both green) and c-FOS (red) (E), and the quantifications of marker-positive cells (D, F, G). **(H)** MEA analysis of Iso. Ctrl and p.Arg493His hCNs showing single-electrode bursts over the period of 29 days after neural induction. *n* = 24. **(I-K)** MEA analysis of Iso. Ctrl and p.Arg493His hCNs showing representative Raster plots of spike distribution on day 29 before and after electrical stimulations (black triangles) (I), the number of network bursts (J), and network inter-burst interval coefficient of variation (IBI-CV) (K) post-stimulation. *n* = 7-17 replicates. **(K)** Body weight measurement of male and female KI mice and their WT littermates at P4, P8, and P30. **(L)** Quantifications of the cytoplasmic-to-nuclear ratio of Celf2 levels in E17.5 WT and KI mouse cortices. *n* = 3 (50 cells each). Nuclei were counterstained with Hoechst 33258. Data are presented as means ± SEM. Each dot represents one experiment or animal. Unpaired t-test (F, L, M), two-way ANOVA with Šídák's multiple comparisons test (H), and Mann-Whitney U test (J, K). Scale bars: 10 µm.

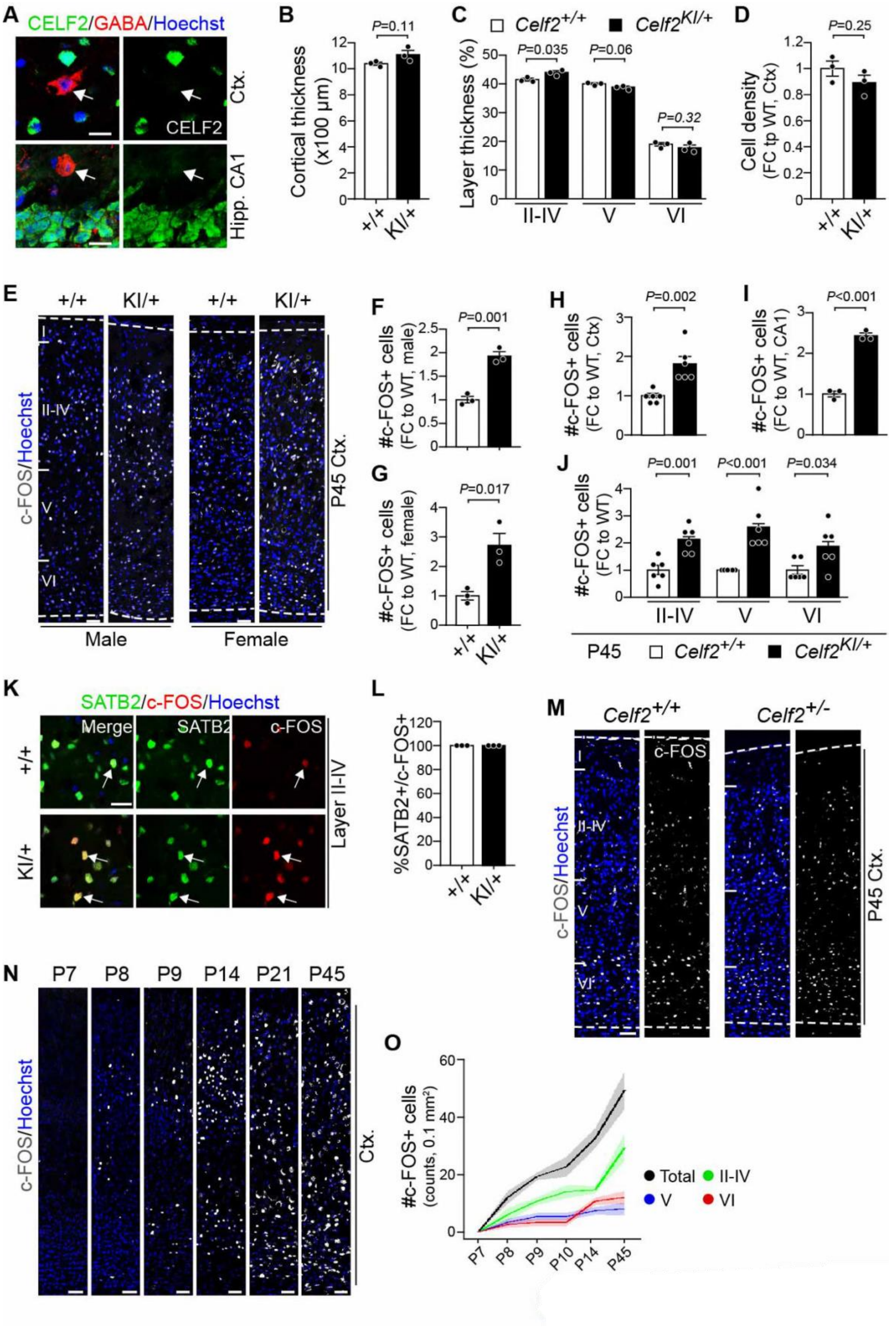
A
CELF2/GABA/Hoechst
Ctx.
CELF2
Hipp. CA1
B
P=0.11
Cortical thickness (x100 µm)
+/+
KI/+
C
Celf2+/+
Celf2KI/+
P=0.035
P=0.06
P=0.32
Layer thickness (%)
II-IV
V
VI
D
P=0.25
Cell density (FC tp WT, Ctx)
+/+
KI/+
E
+/+
KI/+
+/+
KI/+
I
II-IV
V
VI
c-FOS/Hoechst
P45 Ctx.
Male
Female
F
P=0.001
#c-FOS+ cells (FC to WT, male)
G
P=0.017
#c-FOS+ cells (FC to WT, female)
+/+
KI/+
H
P=0.002
#c-FOS+ cells (FC to WT, Ctx)
I
P<0.001
#c-FOS+ cells (FC to WT, CA1)
J
P=0.001
P<0.001
P=0.034
#c-FOS+ cells (FC to WT)
II-IV
V
VI
P45
Celf2+/+
Celf2KI/+
K
SATB2/c-FOS/Hoechst
Merge
SATB2
c-FOS
+/+
KI/+
Layer II-IV
L
%SATB2+/c-FOS+
+/+
KI/+
M
Celf2+/+
Celf2+/-
c-FOS
I
II-IV
V
VI
c-FOS/Hoechst
P45 Ctx.
N
P7
P8
P9
P14
P21
P45
c-FOS/Hoechst
Ctx.
O
#c-FOS+ cells (counts, 0.1 mm²)
Total
II-IV
V
VI
P7
P8
P9
P10
P14
P45

Supplemental Figure 3. Mislocalization of CELF2 rather than its LoF causes neuronal hyperactivity. (A) Confocal images of cortical (Ctx.) and hippocampal (Hipp.) regions from P30 coronal brain sections, immunostained for CELF2 (green) and GABA (red). Arrows indicate GABA-positive inhibitory neurons. **(B-D)** Quantifications of total cortical thickness (B), thickness of cortical layers (C), and cortical cell density (D) in WT and KI mice. **(E-J)** Analysis of P45 mouse cortex immunostained for c-FOS (white, E), showing the number of c-FOS positive cells in male (F) and female (G) KI mice normalized to WT mice, as well as quantifications across the whole cortex (H), hippocampal CA1 region (I), and specific cortical layers (J). **(K)** Confocal images of P45 WT and KI cortex immunostained for SATB2 (green) and c-FOS (red). **(L)** Quantifications of the proportion of c-FOS+ cells co-expressing SATB2+. **(M)** Confocal images of cortical sections from P45 WT and heterozygous *Celf2* KO mice. **(N, O)** Confocal images (N) of cortical sections at successive postnatal ages, immunostained for c-FOS (white) and quantifications of c-FOS-positive cells in cortical layers (O). Nuclei were counterstained with Hoechst 33258. Data are presented as means ± SEM. Each dot represents one animal. Unpaired t-test. Scale bars: 10 μm in (A), 25 μm in (K), 50 μm in (E, M, N).

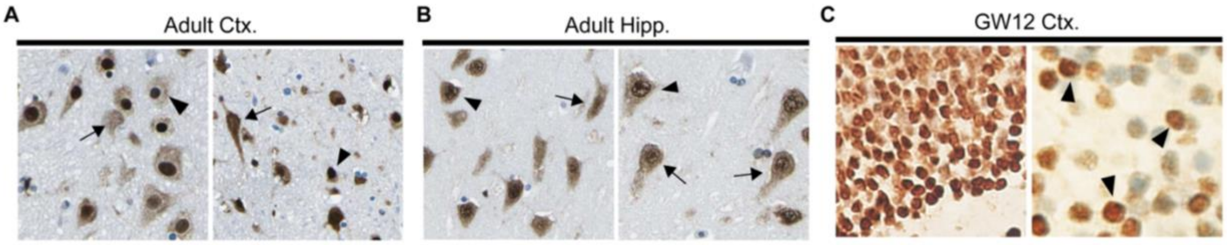

Supplemental Figure 4. CELF2 shows distinct subcellular localization patterns in the embryonic and adult human brains. (A, B) Images from Human Protein Atlas (proteinatlas.org) showing CELF2 expression in the human adult cortex (A) and hippocampus (Hipp.) (B). **(C)** Microscopic images of human cortical (Ctx.) sections at gestational week 14 (GW14), immunostained for CELF2. Arrows highlight cytoplasmic CELF2, while arrowheads denote neurons with greater nuclear localization.

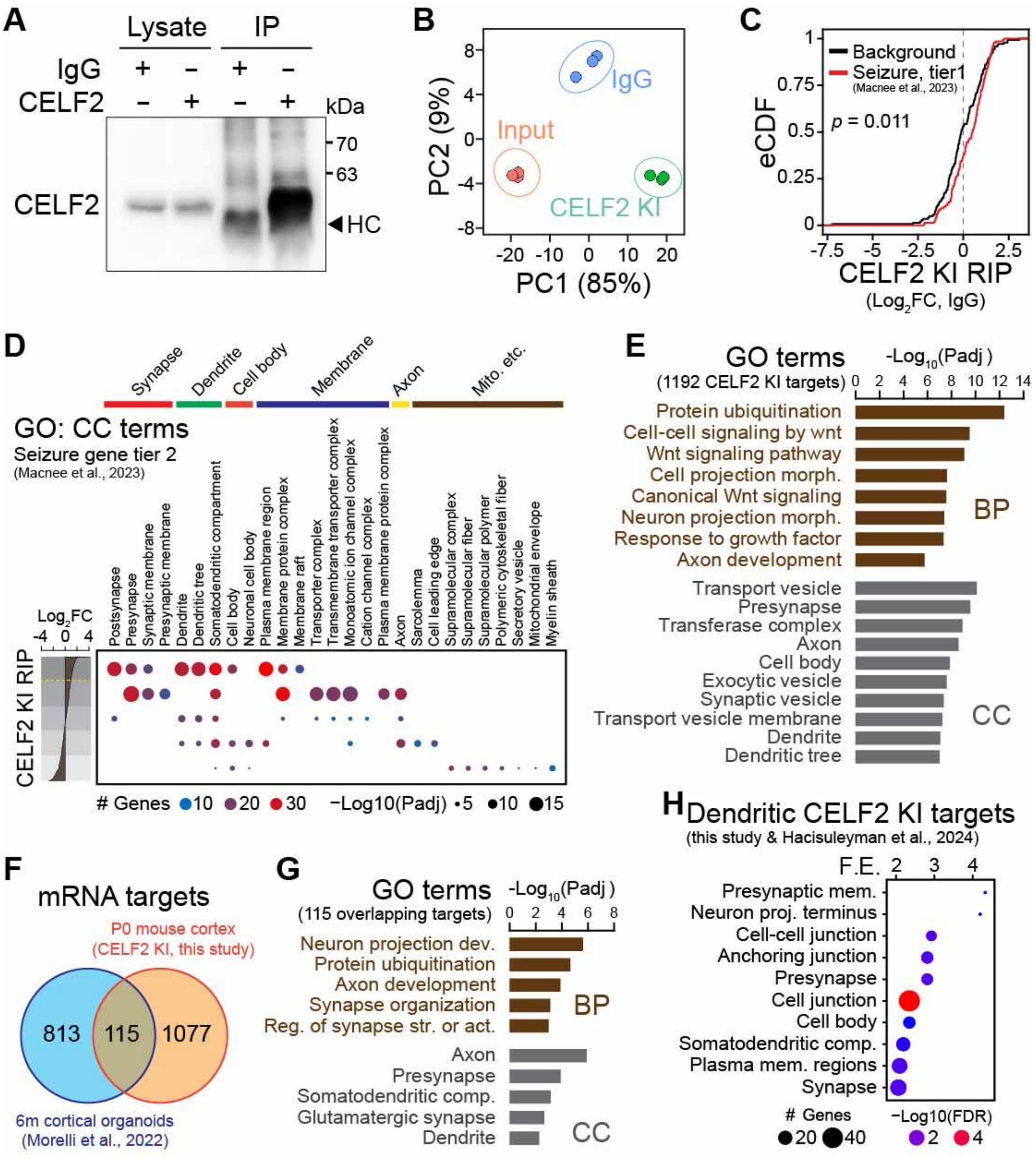

Supplemental Figure 5. RNA-immunoprecipitation-sequencing (RIP-seq) identifies target mRNAs bound by CELF2 p.Arg493His mutant in the cytoplasm. (A) WB of E12.5 KI cortical lysates immunoprecipitated with either control IgG or an anti-CELF2 antibody, probed for CELF2. The arrowhead denotes the IgG heavy chain. **(B)** Principal component analysis (PCA) of RNA-seq samples from input, CELF2 KI RIP, and IgG RIP. **(C)** Cumulative distribution of $\log_2$-transformed fold change (RIP/IgG) for 142 tier 1 seizure-related genes (red) (38) compared to an equal number of randomly selected genes (black). **(D)** Dot plot of enriched GO terms from 514 tier 2 seizure-related genes, grouped into five bins of equal size based on their RIP enrichment, as shown on the left.

The yellow dotted line marks a 2-fold enrichment threshold (RIP/IgG). The enriched GO terms are grouped into six Cellular Component (CC) categories. Color indicates the number of genes mapped to each term, while dot size represents the adjusted *P*-value. **(E)** GO analysis showing the top enriched Biological Process (BP) and Cellular Component (CC) terms for the 1,192 target genes, with corresponding adjusted *P*-values. **(F)** Venn diagram showing the overlap between target genes identified in the mouse cortex (this study) and in human cortical organoids (9). **(G)** GO analysis showing the top enriched BP and CC terms for the 115 overlapping genes identified in (F), with corresponding adjusted *P*-values. **(H)** Dot plot showing the top six enriched GO CC terms, with fold enrichment (F.E.) values, from the analysis of 185 target mRNAs identified as dendritic mRNAs (39).

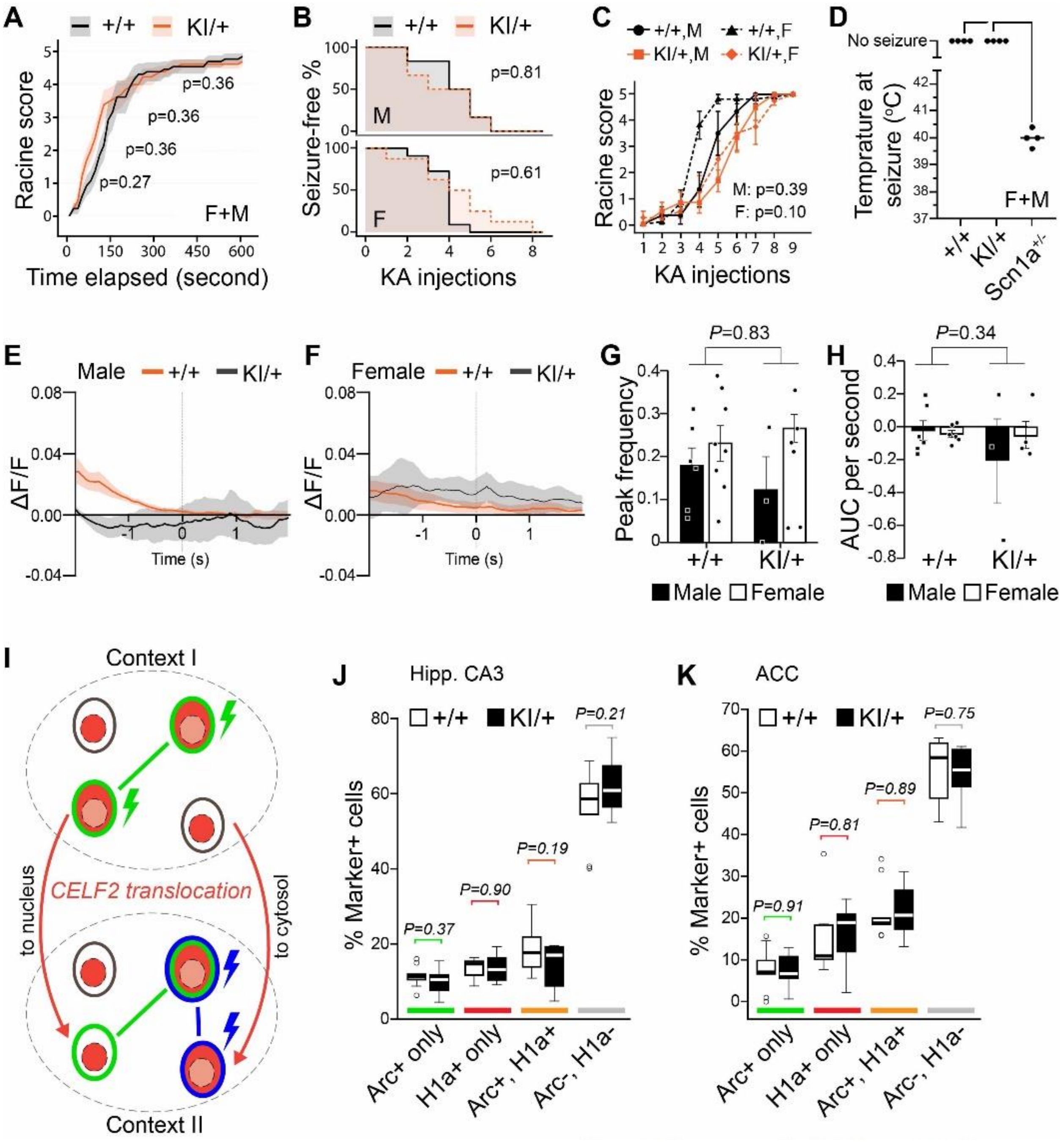

Supplemental Figure 6. Assessment of seizure susceptibility, neuronal activity, and ensemble dynamics in KI mice. (A) Racine seizure score progression following a single PTZ injection in WT and KI mice. Shaded areas represent SEM. Mantel-Cox log-rank test with Benjamini-Hochberg correction. **(B, C)** Analysis of WT and KI male and female mice in the repeated low-dose kainic acid (KA) injection model, showing the percentage of seizure-free mice (B), and Racine seizure score progression (C) following each injection. Log-rank test (B) and Mann-Whitney U test (C) with Benjamini-Hochberg correction. **(D)** Analysis of hyperthermia-induced seizures in WT, KI, and *Scn1a*$^{+/-}$ mice.

One-way ANOVA with Tukey’s post hoc test. Each dot represents one animal. **(E–H)** Photometry traces depicting calcium signal dynamics during transitions from movement to freezing (±2 s from freezing onset) in male (E) and female (F) mice, with no significant differences in peak signal frequency (G) or AUC (H) observed between KI and WT mice in either sex. **(I)** Schematic illustrating neuronal activation (act.) following sequential exploration of Context I and Context II. **(J, K)** Quantifications of IEG expression in neurons within the hippocampal CA3 region (J) and the anterior cingulate cortex (ACC) (K). $n \geq 7$ each. Unpaired t-test. Data are presented as means ± SEM.

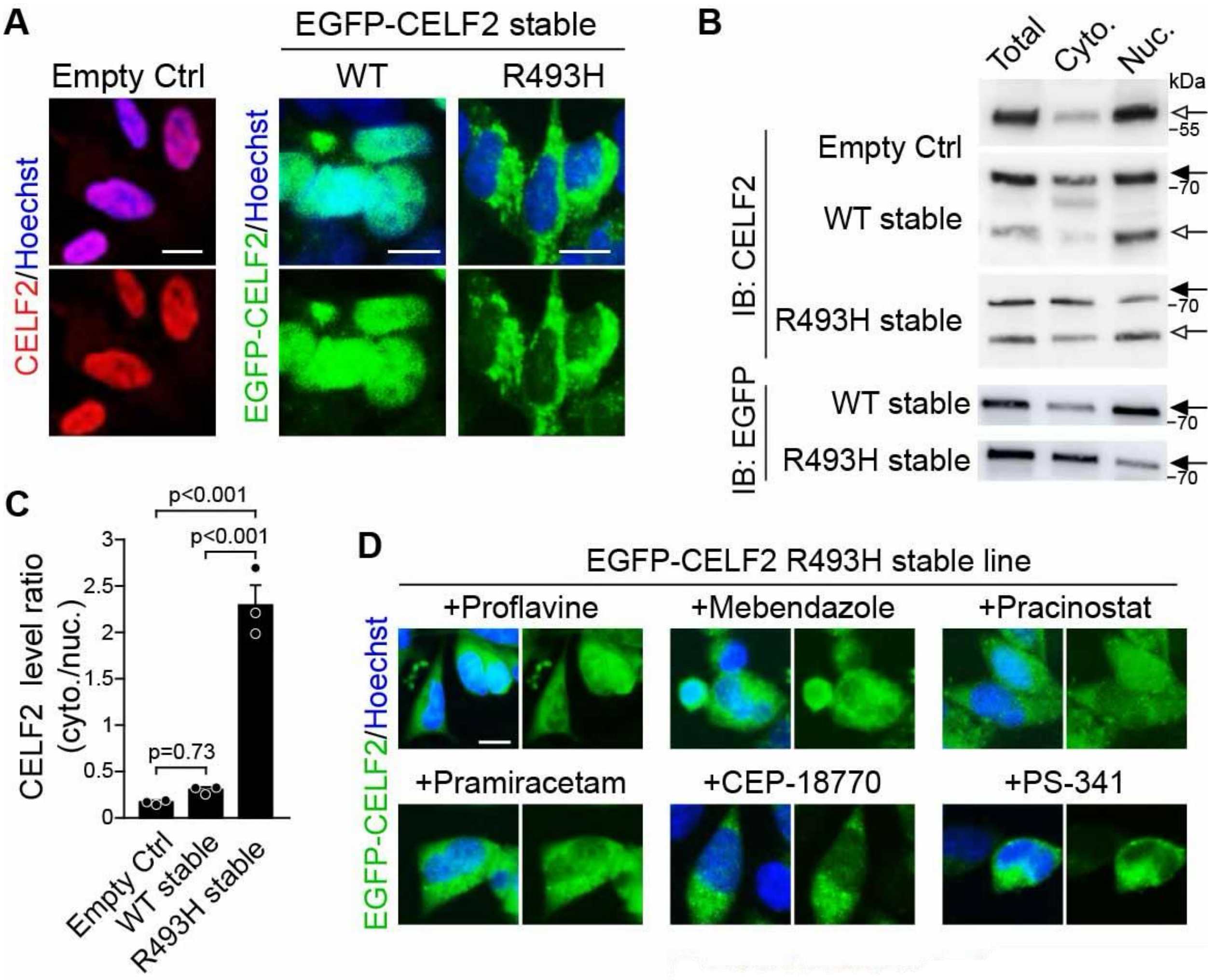

Supplemental Figure 7. Cell-based screening identifies compounds that modulate the localization of EGFP-tagged CELF2 p.Arg493His. (A) Confocal images of HEK293 cells or stable lines expressing EGFP-tagged WT or p.Arg493His mutant CELF2 (green), immunostained for CELF2 (red). **(B)** Western blots of total lysates and cytoplasmic and nuclear fractions from HEK293 cells and stable lines, probed for CELF2 and EGFP. Filled and open arrows indicate EGFP-CELF2 and endogenous CELF2, respectively. **(C)** Quantifications of EGFP-CELF2 subcellular distribution from (B). Data are presented as means ± SEM. One-way ANOVA with Tukey post hoc test. Each dot represents one experiment. **(D)** Confocal images of the HEK293 stable line expressing p.R493H CELF2 (green), following treatment with the indicated compounds. Scale bars: 10 μm.

Supplemental Methods

Animals. All animal use was approved by the Animal Care Committee at the University of Calgary and conducted in accordance with the Canadian Council of Animal Care. For inhibitory neuron visualization, Slc32a1-IRES-Cre driver mice (JAX: 016962) were crossed with Ai14 (JAX: 007914) reporter mice. *Scn1a*$^{+/-}$ mice (C57BL/6J x 129S6/SvEvTac-Scn1atm1Kea F1) were used for hyperthermia seizure experiments.

HEK293 cell culture and stable cell line generation. HEK293 cells (ATCC, CRL-1573) were cultured in DMEM supplemented with 10% FBS and 1% penicillin/streptomycin. Stable HEK293 cell lines expressing EGFP-CELF2 WT or EGFP-CELF2 p.Arg473His were generated using the pInducer20 doxycycline-inducible expression plasmid and a second-generation lentiviral packaging system comprising the pCMV-VSV-G envelope and pCMV-dR8.2 dvpr packaging plasmids. Lentiviral plasmids were transfected into LentiX packaging cells (Takara Bio) using Lipofectamine 3000 (Thermo Fisher Scientific) per the manufacturer's protocol. After 48 hours, medium was collected, filtered through a 0.45 μm syringe filter, and ultracentrifuged. Purified lentiviral particles were resuspended in DMEM and used to transduce HEK293 cells.

Primary mouse neuronal culture. Primary cortical neurons were prepared from E16.5 CD1 mouse embryos (Charles River Laboratories). Cortices were dissected free of meninges, dissociated in 0.05% trypsin-EDTA, and plated on coverslips pre-coated overnight with poly-D-lysine (1 mg/mL) and laminin (5 mg/mL). Neurons were cultured in Neurobasal Medium (Thermo Fisher Scientific) supplemented with 2% B27, 0.5 mM L-glutamine, and GDNF. To suppress glial proliferation, 5 μM cytosine arabinoside (Ara-C) was added on DIV1. Half-medium changes were performed every 2–3 days. For tetrodotoxin (TTX) experiments, TTX (1 μM final concentration) was applied 48 hours before fixation or harvest at DIV13.

Transfection of HEK293 cells and hiPSCs. HEK293 cells were seeded in 12-well plates (minigene reporter analysis), 6-well plates (WB analysis), or on glass coverslips in 24-well plates (immunostaining). At 70-80% confluency, cells were transfected with 0.5-1 ug (24-well), 1-2 ug (12-well), or 3-5 ug (6-well) of plasmid DNA using Lipofectamine 3000 (Thermo Fisher Scientific) according to the manufacturer's instructions. For minigene reporter analysis in hiPSCs, cells were seeded in 12-well plates and transfected using the same protocol with the appropriate DNA amount. Transfection complexes prepared in Opti-MEM were added directly to the culture medium, and cells were incubated for 24 hours before downstream analysis. Transfection efficiency was assessed by fluorescence microscopy based on EGFP expression.

Molecular cloning and plasmid preparation. *CELF*2 variants were generated using the In-Fusion mutagenesis approach using the Snap Assembly kit (Takara Bio). Plasmids were transformed into chemically competent E. coli DH5a cells via heat shock and plated onto LB agar plates containing appropriate antibiotics. Resulting colonies were screened by colony PCR, and positive clones were confirmed by Sanger sequencing to verify correct insertion and orientation.

Minigene reporter preparation and splicing analysis. *CELF*2 variants were introduced into pEGFP-CELF2 WT by mutagenesis (*7*). To assess variants that introduce premature termination codons (PTCs), minigene reporters were constructed containing an ATG-containing EGFP coding sequence fused in-frame to the variant-relevant upstream exon (WT or variant), flanking intronic sequences, and the downstream exon terminated by an in-frame TAA normal termination codon (NTC) (Supplemental Figure 1D). For p.Q34X, exons 2 and 3 were included; for p.Q230X, exons 7 and 8/9 were included; and for G414AfsX45, exons 11/12 and 13 were included. All constructs were synthesized by GeneArt (Thermo Fisher) or VectorBuilder and cloned into pcDNA3 under a CMV promoter. HEK293 cells and hiPSCs were transfected with splicing reporters, and RNA was collected 24 hours later. Relative transcript expression was assessed by qRT-PCR using EGFP normalized to the neomycin resistance gene (Neo), with GAPDH as an additional internal control, and calculated using the ΔΔCt method.

qRT-PCR. HEK293 cells and hiPSCs were seeded in 12-well plates and cultured to 50-60% confluency and transfected as described above. Cells were incubated for 24 hours post-transfection before RNA extraction. Total RNA was extracted using the E.Z.N.A. Total RNA Kit (Omega Bio-tek) according to the manufacturer's instructions. The purified RNA was subsequently reverse transcribed into cDNA using the iScript cDNA Synthesis Kit (Thermo Fisher Scientific), following the manufacturer’s protocol. qPCR was performed using the CFX96 Connect Real-Time PCR Detection System (Bio-Rad) with PerfeCTa SYBR Green FastMix. The amplification protocol consisted of an initial denaturation at 95 °C for 2 minutes, followed by 40 cycles of 94 °C for 15 seconds, 60 °C for 15 seconds, and 72 °C for 15 seconds. Amplicon specificity was confirmed by melt curve analysis consisting of steps at 95 °C for 10 seconds, 65 °C for 5 seconds, and 95 °C for 0.5 seconds. Each sample was analyzed in 2 or 3 technical triplicates, and cycle threshold (Ct) values were averaged for relative quantification. Relative expression levels were calculated as fold changes using the ΔΔCt method.

DNA extraction and PCR. Genomic DNA was extracted from cells and tail tissues for genotyping using the E.Z.N.A. Tissue DNA Kit (Omega Bio-tek) according to the manufacturer’s protocol. Each 10 uL PCR reaction contained 0.5 uL of template DNA, 0.5 uL of each primer, and 5 uL DreamTaq Green PCR Master Mix (2x) (Thermo Fisher

Scientific). PCR amplification consisted of an initial denaturation at 95°C for 3 minutes (1 cycle), followed by 35 cycles of denaturation at 95°C for 30 seconds, annealing at 68°C for 30 seconds, and extension at 72°C for 30 seconds, with a final extension at 72°C for 1 minute. DNA products were resolved by a 1.5% agarose gel electrophoresis.

Cell fractionation. To separate cytoplasmic and nuclear fractions, cells were harvested and pelleted by centrifugation in PBS. For whole-cell lysates, the pellet was resuspended directly in Laemmli buffer (0.25 M Tris-HCl pH 6.8, 8% SDS, 40% glycerol, 0.05% bromophenol blue, and 100 mM DTT). To obtain cytoplasmic and nuclear fractions, cells were resuspended in 0.05% NP-40 Alternative, incubated on ice for 2-3 minutes, and centrifuged at 2,000 x g for 15 seconds. The supernatant was collected as the cytoplasmic fraction. The pellet was washed twice in the same buffer and retained as the nuclear fraction. Both fractions were transferred to separate tubes and mixed with Laemmli buffer. For cortical tissue, dissected cortices were dissociated in trypsin-EDTA for 2-3 minutes, triturated, washed in PBS, and resuspended in NP-40 buffer as described above. All fractions were sonicated twice for 5 seconds each, boiled for 1 minute, and analyzed by WB.

Immunoblotting. Samples were mixed with Laemmli buffer, boiled, and resolved on 4-15% SDS-PAGE gels for 1.25-2 hours. Proteins were then transferred onto nitrocellulose membranes and blocked with 4% skimmed milk powder or bovine serum albumin (BSA) in TBS-T for 1 hour at room temperature. Membranes were probed overnight at 4°C with primary antibodies diluted in blocking solution, followed by incubation with HRP-conjugated secondary antibodies in blocking solution for 1 hour at room temperature. Protein bands were detected using Western blotting detection reagent or SuperSignal West Pico PLUS Chemiluminescent Substrate, imaged on an Amersham Imager 600 (GE Healthcare), and quantified using ImageJ.

Immunocytochemistry. Cultured cells (HEK293, primary mouse neurons, hiPSCs, hNPCs, and hCNs) on 12 mm coverslips were washed with PBS and fixed in 4% PFA in PBS for 10 minutes at room temperature. PFA was removed, and cells were rinsed twice with PBS, then permeabilized with 0.3% Triton X-100 in PBS for 10 minutes. After rinsing with PBS, cells were incubated in blocking buffer (10% normal donkey serum (NDS) and 1% BSA in PBS) for 45-60 minutes at room temperature. Blocking buffer was removed, and coverslips were transferred to a humidified staining chamber. Primary antibodies were diluted 1:1 in blocking buffer and PBS and applied to each coverslip for overnight incubation at 4°C. The following day, coverslips were washed three times with PBS, and appropriate secondary antibodies diluted in PBS were applied and incubated for 1 hour at room temperature. Nuclei were counterstained with Hoechst 33258. Following three additional PBS washes, coverslips were mounted onto

Superfrost Plus microscope slides (Thermo Fisher Scientific) using Aqua-Poly/Mount (PolySciences) and sealed with clear nail polish.

Immunohistochemistry. For immunostaining of embryonic or postnatal cortices, brains were dissected in ice-cold HBSS, fixed in 4% PFA at 4°C for 4 hours, cryopreserved in 30% sucrose, and stored in embedding molds at -80°C prior to cryosectioning. Brains were cryosectioned coronally at 18 μm. For immunostaining of adult cortices, mice underwent transcardial perfusion with PBS followed by 4% PFA, after which brains were dissected and post-fixed in 4% PFA overnight at 4°C, followed by cryopreservation. Brains were cryosectioned coronally at 20 μm. Sections were washed twice in PBS and blocked with 5% BSA and 0.3% Triton X-100 in PBS for 1 hour at room temperature. Sections were then incubated with appropriate primary antibodies in blocking buffer overnight at 4°C, washed three times with PBS, and incubated with appropriate secondary antibodies diluted in blocking buffer for 1 hour in the dark at room temperature. Nuclei were counterstained with Hoechst 33258. For human tissue, cortical sections from 14-gestational-week embryos obtained following elective termination of pregnancy were processed as formalin-fixed paraffin-embedded sections. Sections were deparaffinized in xylene at 60°C for 1 hour and rehydrated through a graded ethanol series (100-50%). Antigen retrieval was performed by boiling sections in 1x sodium citrate buffer at 95°C for 15 minutes. After cooling to room temperature, endogenous peroxidase activity was quenched with 3% hydrogen peroxide in PBS. Sections were then blocked with 5% normal goat serum and 0.3% Triton X-100 in PBS at 37°C for 30 minutes and incubated with anti-CELF2 antibody at 37°C for 1-2 hours. Detection was performed using a rabbit IgG immunohistochemistry kit according to the manufacturer's instructions.

RNA immunoprecipitation (RIP)-sequencing. For RIP-seq, cortices were dissected from P0 heterozygous *Celf2* KI mice, dissociated, and subjected to RIP using the EZ-Magna RIP RNA Immunoprecipitation Kit according to the manufacturer's instructions with modifications. Briefly, the cytoplasmic fraction was prepared as described above and supplemented with protease and RNase inhibitors. An aliquot was reserved for RNA extraction as the input control. The remaining supernatant was precleared with protein A/G beads and incubated with 10 ug of rabbit anti-CELF2 antibody or normal rabbit IgG for 3 hours at 4°C. Beads were washed, and co-precipitated RNAs were isolated for RNA sequencing.

RNA-sequencing and processing. Total RNA was isolated from three biological replicates each of input, IgG control, and CELF2 immunoprecipitates, extracted by phenol/chloroform, and subjected to rRNA depletion using the NEBNext rRNA Depletion Kit. Libraries were prepared using the NEBNext Ultra II Directional RNA Library Prep Kit and sequenced on a NextSeq 500 instrument (Illumina). Sequencing quality was

assessed using FastQC (v0.11.9), and adapters and low-quality bases were trimmed using fastp (v0.23.4). Residual rRNA reads were identified and removed using Ribodetector (v0.3.1). Transcript quantification was performed using Kallisto (v0.51.1) via pseudoalignment against the Mus musculus reference transcriptome obtained from Ensembl, and the resulting read counts were used as input for differential expression analysis.

Differential expression and bioinformatics. Transcript counts were converted to gene-level counts, retaining only protein-coding genes for downstream analysis. Genes with low expression were filtered based on a counts per million (CPM) threshold, and trimmed mean of M values (TMM) normalization was applied. Principal component analysis (PCA) was performed using DESeq2 (v1.46.0) on regularized log-transformed gene-level counts to assess sample reproducibility and distinguish between experimental conditions. Differentially expressed genes (DEGs) were identified using generalized linear models in edgeR (v4.4.0), comparing CELF2 KI immunoprecipitates against input and IgG controls. P-values were corrected for multiple comparisons using the Benjamini-Hochberg method to control the false discovery rate (FDR) at 0.01, and DEGs were selected based on a minimum two-fold change in expression. A total of 1,192 genes were overrepresented in the CELF2 KI immunoprecipitates. Gene ontology (GO) enrichment analysis was performed using g:Profiler with a term size range of 1-2000, revealing associations with biological process (BP), cellular component (CC), and Human Phenotype Ontology (HPO) terms. Heatmaps were generated using z-transformed log-CPM expression values of selected functional genes. DEGs were compared with tier 1 seizure-related genes proposed by Macnee et al. (*38*), identifying 142 overlapping genes. The empirical cumulative distribution function (eCDF) was prepared for these overlapping genes and all DEGs. The 514 overlapping DEGs (tier 2) were classified into five groups based on LFC of CELF2 KI versus IgG. GO analysis of each subgroup was performed using DAVID. DEGs were further compared with dendritic mRNAs from hippocampal CA1 neurons (*39*), yielding 185 overlapping genes that were analyzed using ShinyGO. DEGs were also compared with CELF2 targets identified in human cortical organoids (*9*), revealing 115 overlapping genes, and GO enrichment analysis of these shared genes was performed using g:Profiler with a term size range of 1-2000.

Optogenetic stimulation. Under isoflurane anesthesia, mice were stereotaxically injected with an AAV vector encoding the light-activated ion channel channelrhodopsin-2 (pAAV-Syn-ChR2(H134R)-GFP) into the lateral entorhinal cortex (EC; AP -3.2, ML +3.6, DV -4.6). Two weeks post-injection, mice were connected to a patch cord and received optical stimulation via pulsed blue light (0.4 mW, 20 Hz, 15 seconds per minute) for 5 minutes, remained in their home cage for 90 minutes and then

transcardially perfused with 4% PFA under isoflurane anesthesia. Brains were cryoprotected in sucrose, frozen, and cryosectioned for c-FOS immunohistochemistry.

Seizure induction in mice. Seizure severity was scored using a modified Racine scale: stage 0, no response; stage 1, movement arrest; stage 2, facial clonus and ear twitching; stage 3, head nodding and myoclonic jerks; stage 4, repeated clonus; and stage 5, tonic-clonic seizures with loss of posture (*99*). For pentylenetetrazol (PTZ) experiments, 6-8-week-old mice received a single subconvulsive dose of PTZ (80 mg/kg, subcutaneous) and were monitored for 30 minutes. For repeated low-dose kainic acid (KA) experiments, 5-6-week-old mice were acclimated for 30 minutes prior to receiving KA injections (5 mg/kg, intraperitoneal) every 30 minutes until Racine stage 5 seizures or a maximum of 11 injections. For hyperthermia-induced seizure experiments, P21-28 (3-4 weeks old) C57BL/6J, *Celf2* KI, and *Scn1a*$^{+/-}$ (C57BL/6J x 129S6/SvEvTac-Scn1atm1Kea F1) were placed individually in a chamber under controlled ambient heating regulated by a TCAT-2AC temperature controller (Physitemp Instruments). Core body temperature was continuously monitored using a lubricated rectal temperature probe and raised by 0.5°C every 2 minutes from 37°C until seizure onset or a maximum of 42.5°C. Seizure threshold temperature and latency were recorded. Mice were euthanized at the experimental endpoint in accordance with institutional guidelines.

Fiber photometry during contextual fear conditioning. Surgeries were performed under isoflurane anesthesia, with meloxicam analgesia administered before surgery and for 3 days postoperatively. Using an automated robotic stereotaxic frame (Neurostar), a burr hole was drilled in the skull overlying the hippocampus, and 200 nL of AAV (pGP-AAV-syn-jGCaMP8f-WPRE; AAV1) was injected into hippocampal CA1 (AP -2.18, ML ±2.0, DV -1.4) via a Nanoject III infusion system (Drummond Scientific). An optic fiber (NA 0.37, core diameter 200 µm) was implanted at the injection site, secured with super glue and protected with a black opaque dental acrylic headcap to seal the skull and support the fiber. Mice were allowed to recover for two weeks and habituated to the optic patch cord connection for three days prior to contextual fear conditioning. During acquisition, mice were connected to the optic patch cord, placed in the conditioning chamber, and allowed to habituate for 2 minutes before receiving three 0.5 mA shocks (2-seconds, 1-minute intervals). Mice remained in the chamber for an additional minute before returning to their home cage. Retention was assessed the following day in a 5-minute re-exposure in the conditioning chamber without shocks. Freezing, defined as the absence of movement except respiration, was measured using ANYmaze (Stoelting) behavioral tracking software. Fiber photometry recordings were acquired throughout both the acquisition and retention sessions using a Neurophotometrics FP3002 system controlled by Bonsai software, with alternating excitation at 415 nm (isosbestic control) and 470 nm (GCaMP8f) at 20 frames per second per channel, with both channels calibrated to 50 µW. Behavioural events were time-locked to the

photometry signal via TTL pulse delivery thorugh ANYmaze. Photometry data were analyzed using custom MATLAB scripts. The isosbestic channel was fit to a biexponential decay to correct for photobleaching, and the resulting signal was used to linearly scale the calcium-dependent 470 nm channel. Photometry signals aligned to the onset of bouts of freezing or movement (± 2 seconds) to standardize events.

Context discrimination task and catFISH analysis. Mice were habituated to handling for 5 days before sequential exposure to two distinct contexts: a white circular open field (context I) and a black square open field (context II) for 5 minutes each, separated by a 20-minute interval in their home cage. Mice were returned to the home cage for an additional 5 minutes before sacrifice. Brains were embedded in OCT, frozen in chilled isopentane (-70°C), cryosectioned coronally (16 μm), and processed for catFISH. *Arc* and *Homer1a* mRNA were detected using the RNAscope Multiplex Fluorescent Assay (Advanced Cell Diagnostics) per the manufacturer's protocol, along with Hoescht nuclear counterstain. Neuronal activation was analyzed by assessing the subcellular localization of *Arc* and *Homer1a* mRNA, with intranuclear puncta interpreted as transcriptional activity induced by context I, and cytoplasmic puncta as activity from context II. The number of *Arc*- or *Homer1a*-positive puncta in the nuclear and cytoplasmic compartments of individual cells were quantified in the CA1, CA3, and ACC regions of the brain using FIJI.

MEA analysis. hNPCs were seeded onto 24-well MEA plates (Axion Biosystems) and differentiated into neurons as described above. Signals were recorded from 16 electrodes per well on the Maestro platform (Axion Biosystems) using Axis Navigator software, with plates equilibrated at 5 minutes at 37°C before acquisition. Spontaneous network activity was recorded daily from DIV14 to DIV28, and weighted mean firing rate, burst number, and burst frequency, were quantified. On DIV29, electrical stimulation was applied to assess evoked network responses (*98*). Baseline spontaneous activity was recorded for 5 minutes prior to biphasic voltage stimulation (10 bursts, 2-second inter-burst intervals, 10 pulses per burst, 10 μs pulse duration, 10 ms inter-pulse interval, ±1V) followed by 10 minutes of post-stimulus recording. Electrodes with ≥5 spikes/min were considered active, and data were averaged across active electrodes, and wells. Mean firing rate, burst frequency, and burst duration were quantified at both the single-electrode and network levels. Single-electrode bursts were detected using an inter-spike interval (ISI) threshold of a minimum 4 spikes per burst and maximum ISI 100 ms. Network bursts were defined by a minimum of 50 spikes, maximum ISI 100 ms and ≥35% participating electrodes. Evoked responses were analyzed from a 10-second peri-stimulus window (1ms binning). Synchrony was quantified using cross-correlogram analysis with a 20 ms post-stimulus window. All parameters were applied uniformly across genotypes.

High-throughput drug screening. EGFP-CELF2 p.Arg473His HEK293 cells were seeded at 20,000 cells per well in black optical 96-well plates and induced with doxycycline (1 µg/mL) overnight and treated with compounds from an FDA-approved drug library (Selleckchem; 1 µM final concentration, 1% DMSO) for 24 hours. Cells were stained with NucBlue (Thermo Fisher), fixed in 4% PFA, and imaged using an InCell 6000 automated confocal microscope (Cytiva) at 40x magnification across 12 fields and 3 Z-slices (2 µm separation, 36 images/well). Wide-field images were acquired using a 488 nm laser (FITC 525/20 nm filter, 3-second exposure) for EGFP, and a 405 nm laser (DAPI 455/50 nm filter, 1-second exposure) for NucBlue at 100% laser power. Brightfield images were captured with a 0.06-second exposure. Images were analyzed using FIJI (ImageJ), and Mander's colocalization coefficient (M2; fraction of Hoescht-positive pixels overlapping EGFP signal) was calculated from a minimum of 6 images per drug per replicate across duplicate screens. Candidate compounds were selected based on screening outcomes and validated in hiPSCs. Compounds were dissolved in DMSO (100 µM stock) and diluted in mTeSR1 medium to working concentrations of 200 pM to 5 µM based on published values, with cytotoxicity assessed using the CytoTox 96 Non-Radioactive Cytotoxicity Assay Kit (Promega). hiPSCs grown to 50-60% confluency on Matrigel-coated 24-well plates containing 12 mm glass coverslips were treated for 24 hours with the appropriate drug, vehicle, or medium-only control prior to fixation, staining, and imaging. Subcellular localization of CELF2 was analyzed using FIJI as described above.

Microscopy and quantification. Z-stack images of immunostained cells or cortical sections were acquired using an Olympus FV3000 confocal microscope with 20x, 40x, or 60x objectives and projected as sum intensity projections using ImageJ. To quantify CELF2 subcellular distribution, a line was drawn from the center of the nucleus to the cytoplasmic border using the Straight-Line tool in ImageJ, and a plot profile was generated for each channel. The cytoplasmic-to-nuclear ratio of CELF2 fluorescence intensity was calculated using Hoechst staining to define nuclear boundaries. For cell cultures, representative images were selected from at least three independent experiments. For brain section analysis, three to four anatomically matched sections per brain from at least three embryos, postnatal, or adult animals per group were imaged at 20x or 60x magnification. For c-FOS quantification and CELF2 intensity measurements, each matched mouse brain section was divided into equal-sized bins of fixed dimensions prior to quantification. For analysis of CELF2 localization at the subcellular level, Z-stack images were acquired at 60x magnification on the FV3000 confocal microscope (Olympus) and projected as sum intensity projections in ImageJ. Images were processed using FluoView software (Olympus), ImageJ, and Adobe Photoshop. The VZ, SVZ, and CP were defined using PAX6, TBR2, and Hoechst staining, respectively.
