# Supplementary material for: Pathological disruption of CELF2 shuttling causes neuronal hyperactivity, learning deficits, and seizures
Source: J Clin Invest. 2026 Jun 11;136(14):e199698. doi: 10.1172/JCI199698 (PMC13367967; doi:10.1172/JCI199698)
Supplement: Unedited blot and gel images [file jci-136-199698-s127.pdf]

Full unedited blot for **Figure 2C**

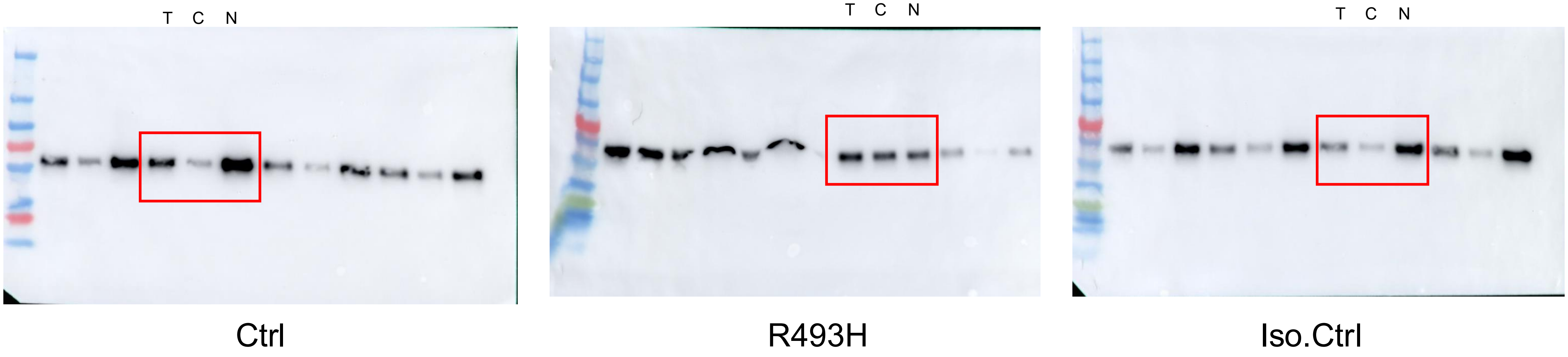

Full unedited blot for **Figure 2N**

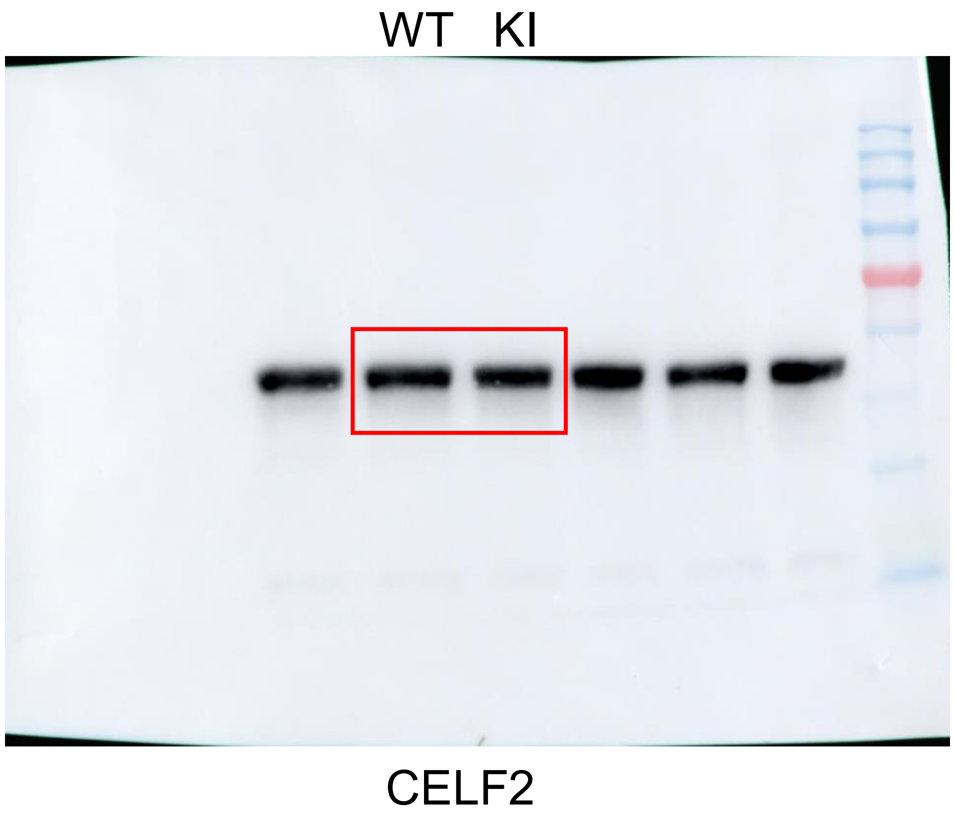

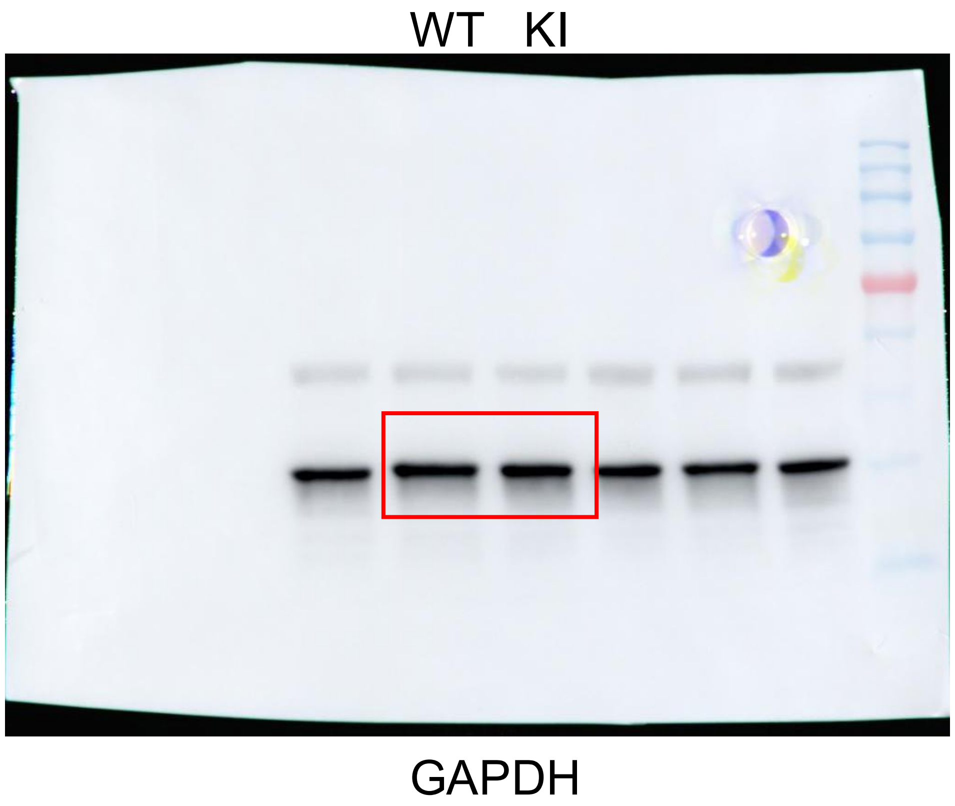

Full unedited blot for **Figure 3I**

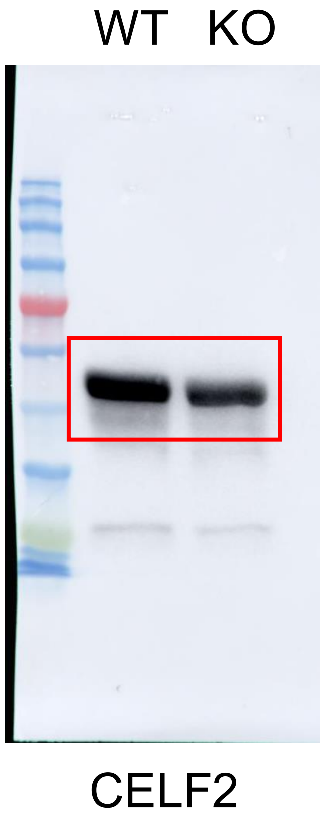

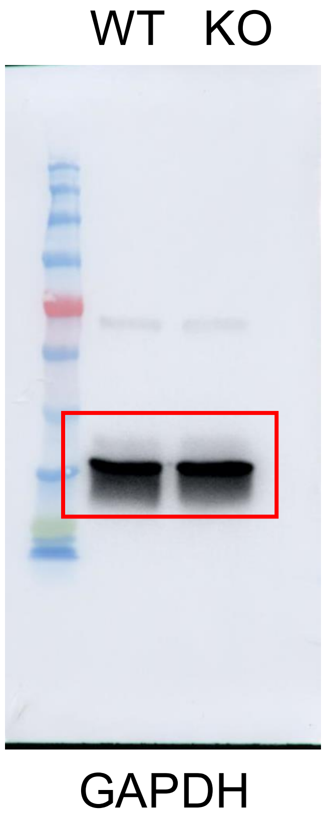

Full unedited blot for **Figure 4D**

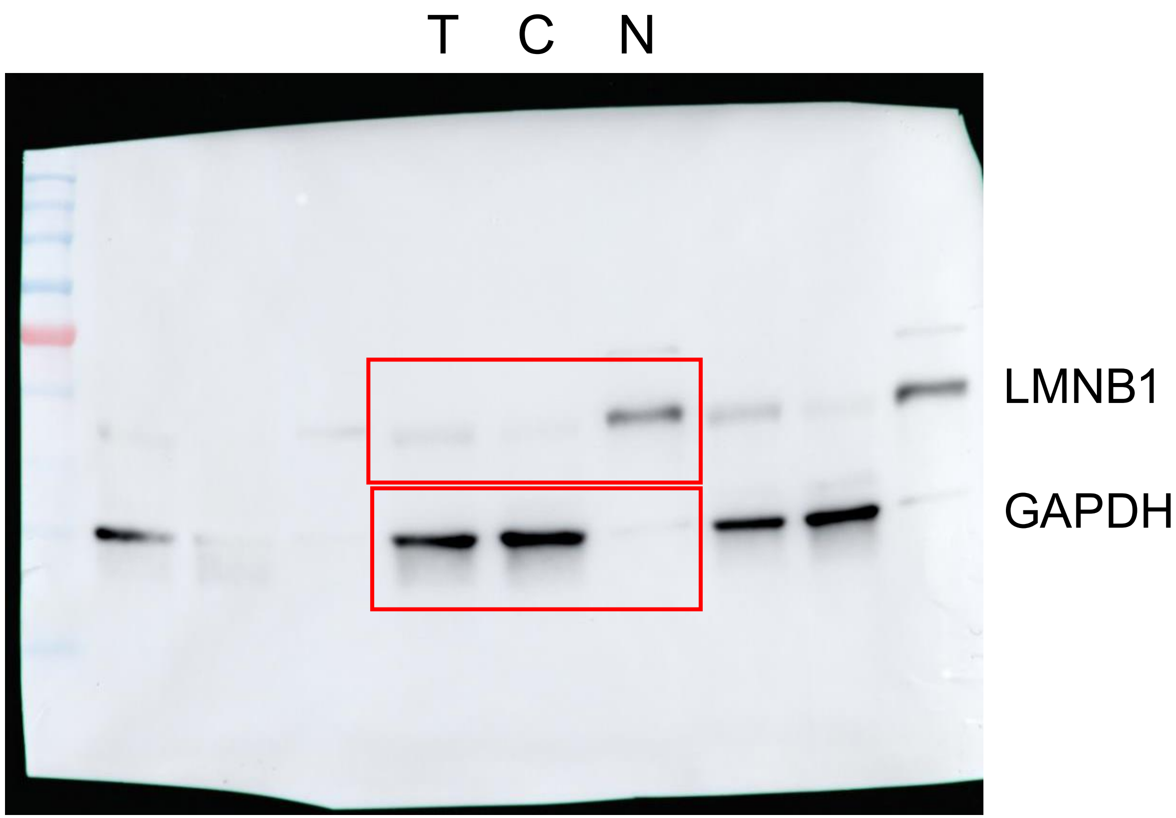

Full unedited blot for **Figure 4E**

Cytoplasmic

P0 P7 P0 P7 P0 P7

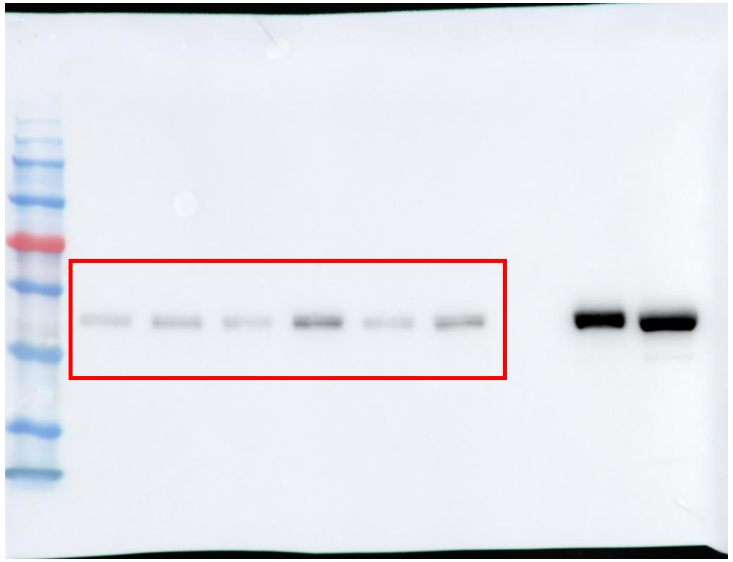

CELF2

Nuclear

P0 P7 P0 P7 P0 P7

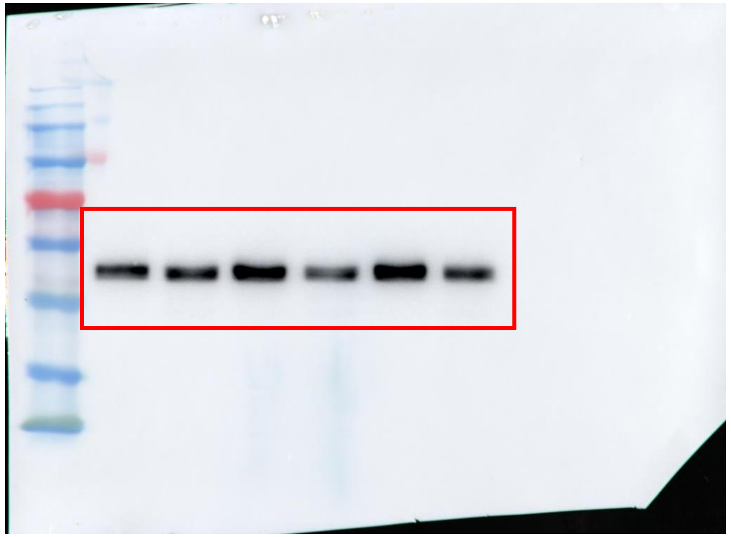

CELF2

Cytoplasmic

P0 P7 P0 P7 P0 P7

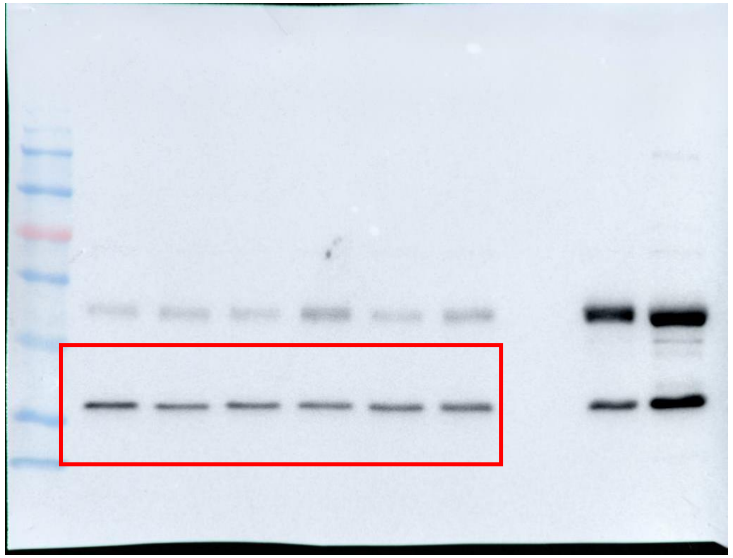

GAPDH

Full unedited blot for **Supplemental Figure 1E**

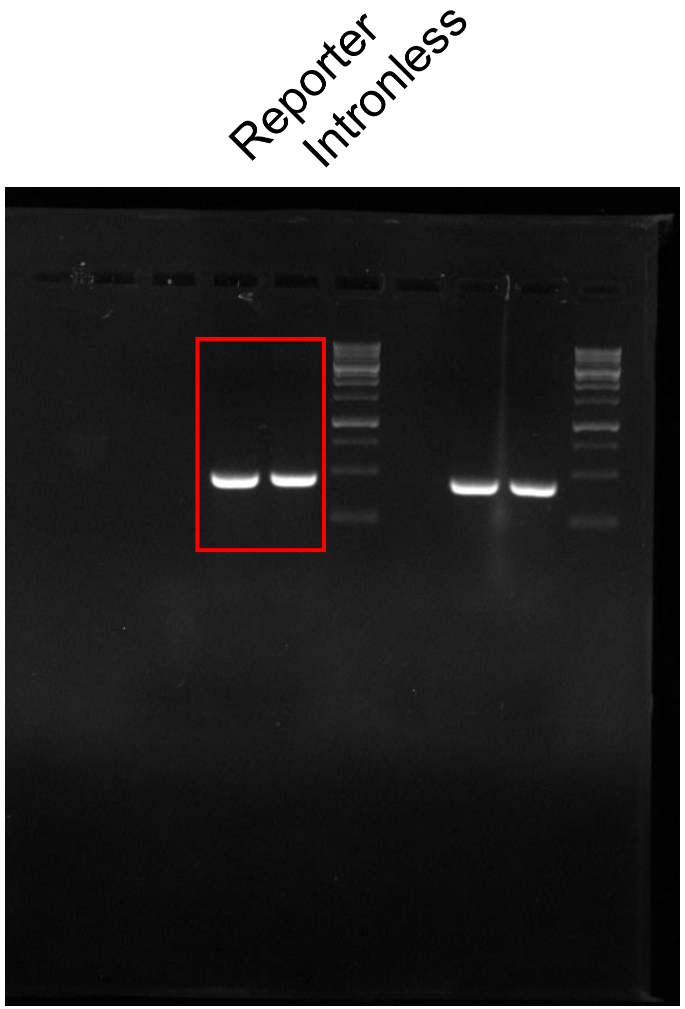

Full unedited blot for **Supplemental Figure 2B**

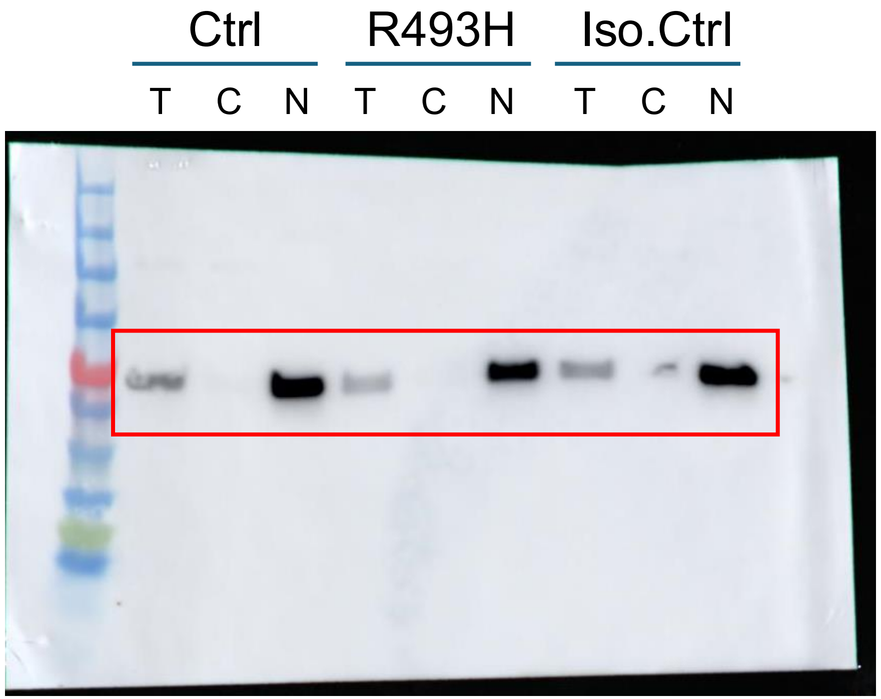

LMNB1

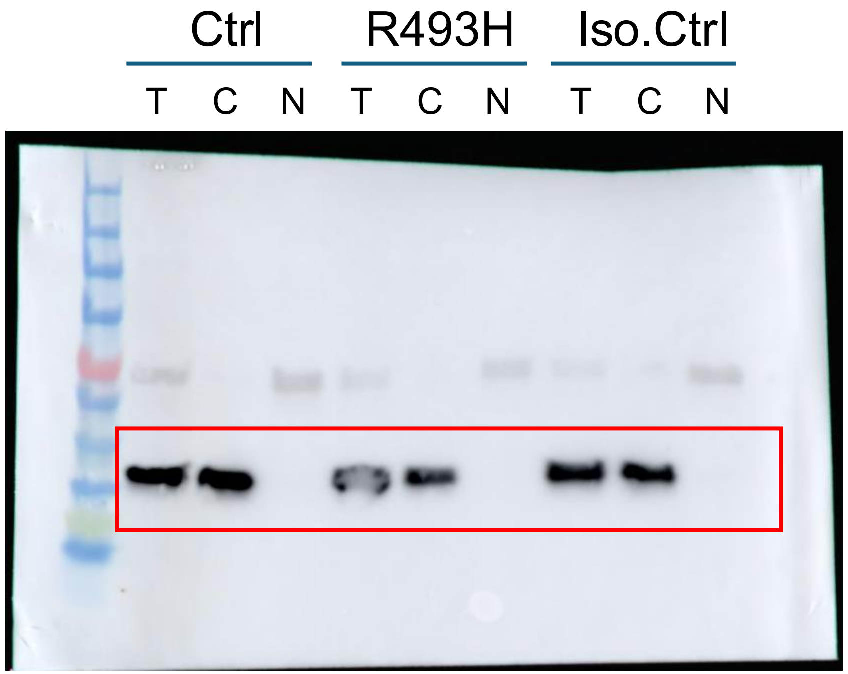

GAPDH

Full unedited blot for **Supplemental Figure 5A**

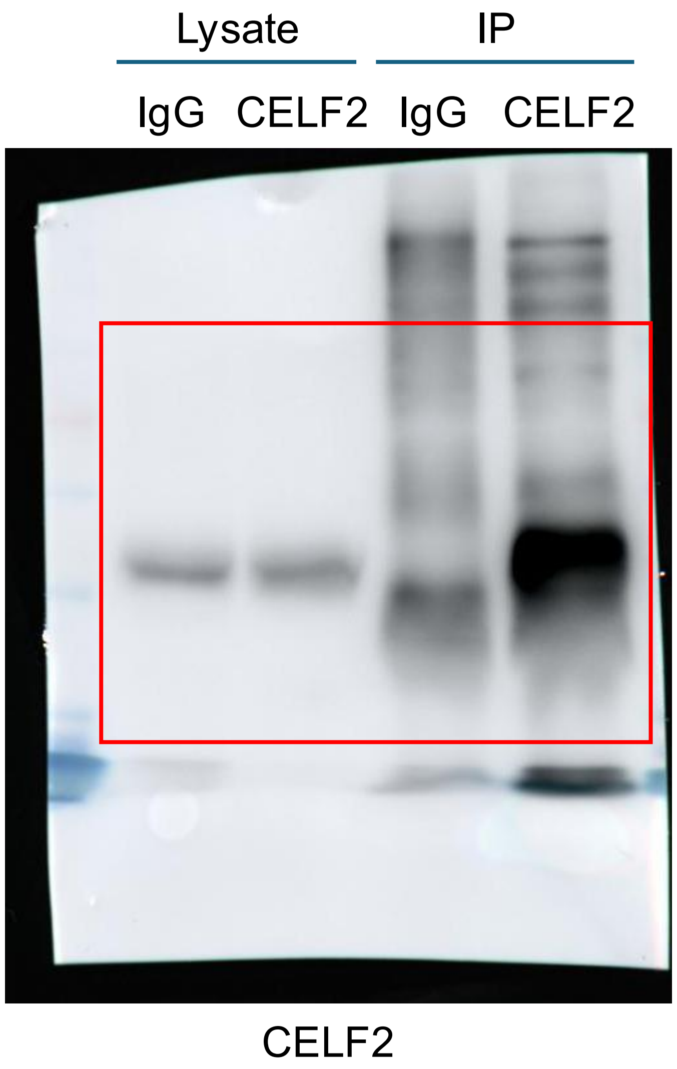

Full unedited blot for **Supplemental Figure 7B**

Empty Ctrl
T C N

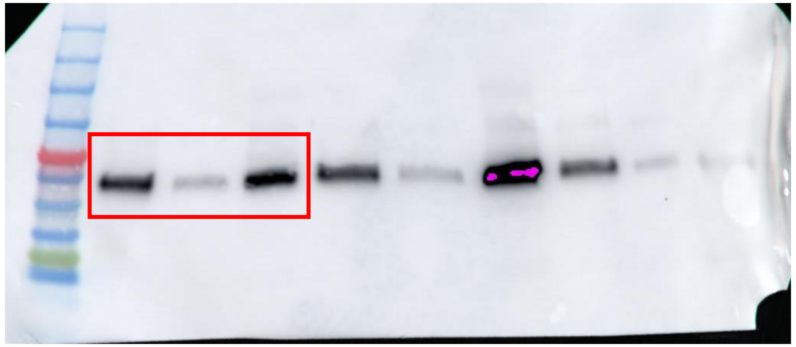

CELF2

R493H stable
T C N

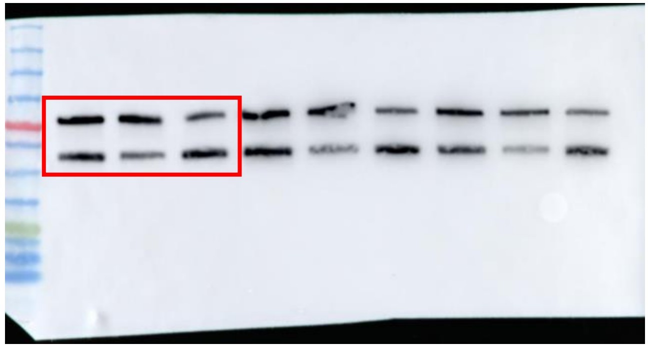

CELF2

WT stable
T C N

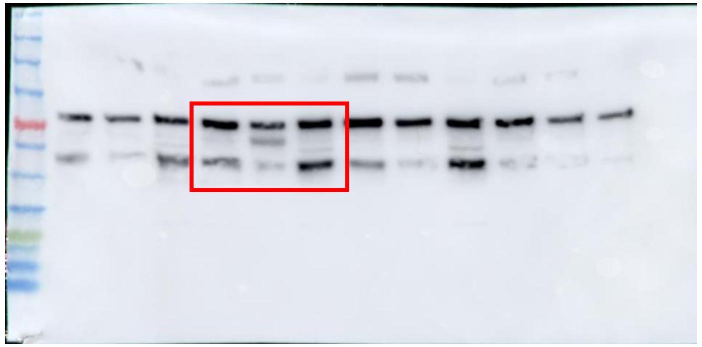

CELF2

WT stable
T C N

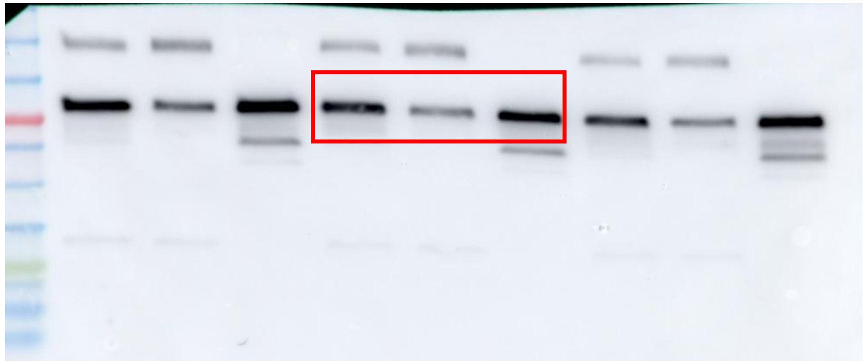

EGFP

R493H stable
T C N

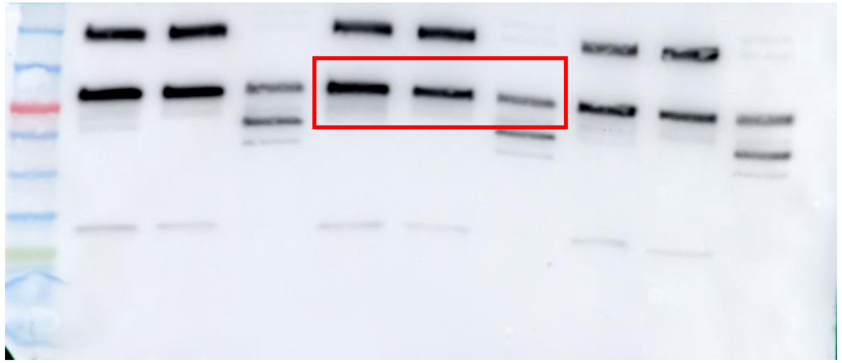

EGFP
